# Polymeric sheet actuators with programmable bioinstructivity

**DOI:** 10.1073/pnas.1910668117

**Published:** 2020-01-13

**Authors:** Zijun Deng, Weiwei Wang, Xun Xu, Oliver E. C. Gould, Karl Kratz, Nan Ma, Andreas Lendlein

**Affiliations:** ^a^Institute of Biomaterial Science, Helmholtz-Zentrum Geesthacht, 14513 Teltow, Germany;; ^b^Berlin-Brandenburg Centre for Regenerative Therapies, Helmholtz-Zentrum Geesthacht, 14513 Teltow, Germany;; ^c^Institute of Chemistry and Biochemistry, Freie Universität Berlin, 14195 Berlin, Germany;; ^d^Institute of Chemistry, University of Potsdam, 14469 Potsdam, Germany;; ^e^Helmholtz Virtual Institute–Multifunctional Materials in Medicine, 14513 Teltow, Germany

**Keywords:** reversible shape-memory actuator, mesenchymal stem cells, calcium influx, HDAC1, RUNX2

## Abstract

Stem cells are capable of sensing and processing environmental inputs, converting this information to output a specific cell lineage through signaling cascades. Despite the combinatorial nature of mechanical, thermal, and biochemical signals, these stimuli have typically been decoupled and applied independently, requiring continuous regulation by controlling units. We employ a programmable polymer actuator sheet to autonomously synchronize thermal and mechanical signals applied to mesenchymal stem cells (MSCs). Using a grid on its underside, the shape change of polymer sheet, as well as cell morphology, calcium (Ca^2+^) influx, and focal adhesion assembly, could be visualized and quantified. This paper gives compelling evidence that the temperature sensing and mechanosensing of MSCs are interconnected via intracellular Ca^2+^. Up-regulated Ca^2+^ levels lead to a remarkable alteration of histone H3K9 acetylation and activation of osteogenic related genes. The interplay of physical, thermal, and biochemical signaling was utilized to accelerate the cell differentiation toward osteogenic lineage. The approach of programmable bioinstructivity provides a fundamental principle for functional biomaterials exhibiting multifaceted stimuli on differentiation programs. Technological impact is expected in the tissue engineering of periosteum for treating bone defects.

## Significance

Stem cells can be conceptualized as computational processors capable of sensing, processing, and converting environmental information (input) to yield a specific differentiation pathway (output). In this study, we employ a temperature-controlled polymer sheet actuator to interpret and transfer information, controlled by the material’s programming, to mesenchymal stem cells. The cell’s interpretation of mechanical, thermal, and biochemical signaling is shown to be dependent on the actuator’s activity, utilized to accelerate differentiation toward bone cells, further elucidating the role of microenvironmental parameters on mammalian cells. Our method provides a unique approach to processing two discrete stimuli into one biochemical signal, calcium ions, providing a basis for the logical control of the flow of biological signals and the design of cellular functions.

In bioinformatics, the mass collection and mass distribution of biological information has enabled the unparalleled collaborative examination of complex cellular systems, detecting previously hidden patterns in the behavior of a variety of organisms. Current cell-signaling methods, however, rely on bulky controlling devices that regulate the transmission of decoupled thermomechanical information, limiting our control of the cellular microenvironment, but because of their expense, also the standardization of protocols that comes with widespread adoption by the scientific community (1, 2). Programmable materials are needed that enable the encoding of information into cell culture substrates, allowing multiple physical signals or inputs to be communicated to cells controllably and autonomously. To effectively replicate the complex microenvironment occupied by in vivo mammalian cells, multiple dynamic inputs are necessary that can be separately controlled and simultaneously applied (3). This allows the differentiation pathway of the cell, or the output, to be modified. Additionally, how the cell senses, processes, and interconnects complex physical signals from its microenvironment is not well understood. Transmitted physical signals are converted by the cell into biochemically relevant information, allowing the regulation of cell behavior. Despite the synergistic nature of this relationship, the roles of mechanical, thermal, and biochemical signaling have traditionally been decoupled and studied separately. Furthermore, methods that allow the in situ analysis of the cells’ shape change during stimulation are needed.

Mesenchymal stem cells (MSCs), a type of multipotent stem cell, are highly sensitive to physical characteristics of their microenvironment such as temperature, differentiated stiffness stress, topography, and extracellular matrix mechanics (4). A range of physical stimuli are able to influence cell behavior, allowing control over lineage commitment (5). The activity of MSCs can be steered via various mechanosignals, as physical cues are sensed via surface receptors such as integrin, and intracellular components can be activated via a mechanotransduction pathway such as Yes-associated protein (YAP) and transcriptional coactivator with PDZ binding domain (TAZ) signaling (6–9). As one of the most important environmental interventions, temperature change has a profound effect on an animal’s physiological response. The reduction of core body temperature extends life span in invertebrates (10), African killifish (11), and even mice (12). Exposure to low temperature can slow the process of cellular energy metabolism. The lineage commitment of stem cells can be regulated by local temperature. For example, culturing MSCs under hypothermal conditions (32 °C) drives cell differentiation into beige-like adipocytes (13). An increased Schwann-like cell differentiation of MSCs was found when culturing at 35 °C (14). When MSCs were exposed to a cyclic cold stress (18 °C for 12 h/d), they preferentially differentiated into the osteogenic lineage (15). Temperature change can be sensed via thermosensitive ion channels in the cell membrane, such as certain transient receptor potential (TRP) channels, and further influences calcium (Ca^2+^) influx (16, 17). However, how mechanical and thermal inputs are sensed and processed simultaneously, or the nature of the biochemical signaling responsible for regulating these pathways is poorly understood.

**Fig. 1.**
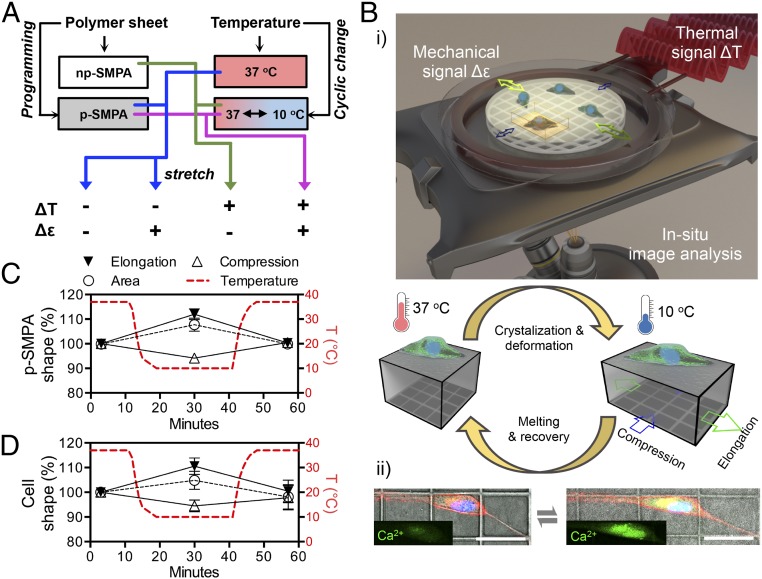
Temperature-controlled p-SMPA sheet exerts synchronized thermal and mechanical stimuli on hADSCs. (*A*) To ascertain the role of each input in logical regulation of hADSCs, cells cultured on SMPA sheets were compared to those exposed to either a constant 37 °C and mechanical stimulation, or a constant temperature and no mechanical stimulation. (*B*) (*i*) Schematic illustration showing the generation of mechanical and thermal signals. The 50 × 50-µm grids on the underside of the sheet enabled the real-time monitoring of the movement of material and cells. (*ii*) Representative images of p-SMPA actuation-induced cell shape change and Ca^2+^ influx showing cytoplasmic membrane (red) and nucleus (blue) and intracellular Ca^2+^ (green). (Scale bars, 50 µm.) (*C* and *D*) Measurements of the temperature-controlled cyclic shape change of the p-SMPA sheet (*C*), and hADSCs cultured on top (*D*), in the direction of elongation and compression, and the change in surface area. The cells were cultured in growth medium on a p-SMPA sheet exposed to cyclic temperature change for 1 d, quantification based on six cells from three randomly selected p-SMPA sheets.

To explore these questions, this study used programmable shape-memory polymer actuator (SMPA) sheets, capable of reversibly and responsively changing between two different predefined shapes (actuation) (18–21). SMPAs are unique among shape-changing materials in that their actuation can be dictated during an initial thermomechanical programming process. In this initial programming period, information is encoded into the material that governs its reaction to an external stimulus. Within the material, crystallizable actuation domains provide the driving force for shape change, while geometry-determining domains provide the network anisotropy necessary to coordinate the change in macromolecular orientation during crystallization and melting (19, 21). We hypothesize that by programming the SMPA sheet it is possible to logically couple thermal and mechanical stimuli synchronously, and use this active substrate to regulate the behavior of human adipose derived stem cells (hADSCs) (Fig. 1*A*). A cyclic temperature change (ΔT) results in a bidirectional 2D actuation (Δε) of the programmed SMPA (p-SMPA), transmitting both thermal and mechanical stimuli (ΔT+, Δε+) to the cells (Fig. 1*B*). For a nonprogrammed SMPA (np-SMPA), a cyclic temperature change generates only a thermal signal (ΔT+, Δε−). An np-SMPA sheet, periodically deformed by a stretch chamber, is used to produce a mechanical stimulus without a cyclic temperature change (ΔT−, Δε+). Finally, a p-SMPA held at a constant temperature generates neither thermal nor mechanical stimuli (ΔT−, Δε−).

Here, a temperature decrease from 37 to 10 °C leads to a simultaneous elongation of the sheet in the direction of deformation during programming, and compression in the perpendicular direction. The unstretched shape can then be recovered by increasing the temperature to 37 °C again (Fig. 1*B*). This cyclic actuation of the p-SMPA drives the shape change of the cells cultured on it, immediately leading to cell response. By molding 50 × 50-µm grids on the underside of the sheet, the accurate real-time visualization of the microscale movement of materials and cells is possible, without influencing the cells cultured on the surface of the sheet. We hypothesize that cyclic temperature-induced shape change of the p-SMPA sheet results in the synchronized shape change of cells. These dual stimuli activate Ca^2+^ signaling, leading to the enhancement of Ca^2+^ influx. YAP signaling is activated by mechanical stimulation but suppressed by Ca^2+^ influx. Acetylation at histone H3 lysine 9 (H3K9ac) on osteogenesis-related gene promoters is promoted by dual stimuli, enhancing osteogenesis and suppressing adipogenesis of hADSCs.

## Results and Discussion

### Synchronization of the Cyclic Shape Change of p-SMPA Sheets and hADSCs.

In this work, semicrystalline linear poly(ε-caprolactone) (PCL) polymers were used as starting materials for the preparation of the polymer networks forming the actuating sheets. The low and broad melting transition (25–50 °C), relatively slow degradation rate, and commercial viability make these materials attractive candidates for many biomedical applications. Its elastic deformation in the temperature range 10–37 °C fulfills the requirements of this study (20). The shape switching temperatures were set within the viability range of the cells between 37 and 10 °C, with a cycle time of 60 min. Although hADSCs showed a detectable cell death when subjected to 10 °C for more than 6 h (*SI Appendix*, Fig. S4*A*), a high cell viability over 7 d (>80%) was sustained under cyclically changed temperature (*SI Appendix*, Fig. S4 *B* and *C*), suggesting the 37 °C temperature (22 min per cycle) allowed for cell recovery. For each cycle, elongation of p-SMPA sheets occurred in the direction of deformation during programming during cooling, with compression occurring in the perpendicular direction, and an overall increase of the material surface area (Fig. 1*C* and *SI Appendix*, Fig. S1*A* and Movie S1). The average elongation of the p-SMPA at 10 °C in cell culture medium was measured as 13.6 ± 0.8% after 24 temperature cycles (day 1), while the np-SMPA displayed an elongation of less than 1% with no distinguishable difference in both macroscopic shape and grid dimensions (*SI Appendix*, Fig. S1*B* and Movie S2).

To verify the long-term behavior of the p-SMPA sheets, necessary to facilitate stem-cell differentiation, their shape change in cell culture medium was recorded for up to 3 wk, with more than 500 temperature-change cycles. An elongation >10% was still observed after 3 wk (*SI Appendix*, Fig. S1*B*). The uniformity of the elongation of the grid squares at different locations of the p-SMPA sheet suggested homogeneous actuation during temperature change (*SI Appendix*, Fig. S1*C*).To ensure that the shape change of the sheet was sufficiently fast relative to the migratory movement of the cells, a temperature program was used to cool from 37 to 10 °C in 8 min and heat from 10 to 37 °C in 8 min. This greatly increased the speed of the shape change of the sheet (*SI Appendix*, Fig. S1*D*). By measuring the change in the distance of two points initially separated by 100 µm, the speed of elongation and compression was measured as 3.6 ± 2.6 µm/min (temperature decrease) and (5.6 ± 1.5) μm/min (temperature increase). The movement speed in the compression direction was measured 2.4 ± 2.2 μm/min (temperature decrease) and 2.7 ± 1.5 μm/min (temperature increase) (*SI Appendix*, Fig. S1*E*). The migration velocity of hADSCs on SMPA sheets was ∼0.6 ± 0.3 µm/min, as quantified via time-lapse microscopy using a previously reported method (22). Therefore, the p-SMPA shape change was sufficiently fast to counteract the migration of hADSCs, which have an average dimension around 100 µm in length, allowing them to feel the mechanical stimulus.

We found no evidence that the presence of cells affected the shape change of the p-SMPA sheets (*SI Appendix*, Fig. S1*F*). This is supported by work elsewhere (23, 24), where the force exerted by cells on a surface has been shown to be more than an order of magnitude less than that of comparable SMPA sheets.

The effect of the actuation on morphology of cells was determined by measuring their change of dimensions during shape change. The cells were stretched and compressed by the shape change of p-SMPA, regardless of their orientation on the surface (*SI Appendix*, Fig. S1 *G* and *H* and Movies S3 and S4). The changes in several key spatial cell parameters, such as the area covered and the length in the direction of elongation and compression, were similar to that of the p-SMPA sheet (Fig. 1 *C* and *D*), suggesting the shape change of the cells was synchronized with the material shape change.

Given that the magnitude of the material shape change is dependent on direction, with a greater shape change in the direction of elongation, cyclic actuation of the sheets should influence the cell orientation. To verify this, cells were seeded at a lower density to ensure the sufficient freedom of movement. After 3 d (72 cycles of actuation), the cells on the p-SMPA sheet aligned preferentially along the direction of elongation, when compared to those on a np-SMPA sheet (*SI Appendix*, Fig. S2). Furthermore, the influence of both stimuli on cell proliferation was assessed. The cell proliferation was inhibited by cyclic temperature change but promoted by mechanical stimulus, as evidenced by the staining of cell proliferation marker Ki67 as well as the quantification of cell number (*SI Appendix*, Fig. S3).

### Regulation of the Ca^2+^ Dynamics of hADSCs by Thermal and Mechanical Dual Stimuli.

**Fig. 2.**
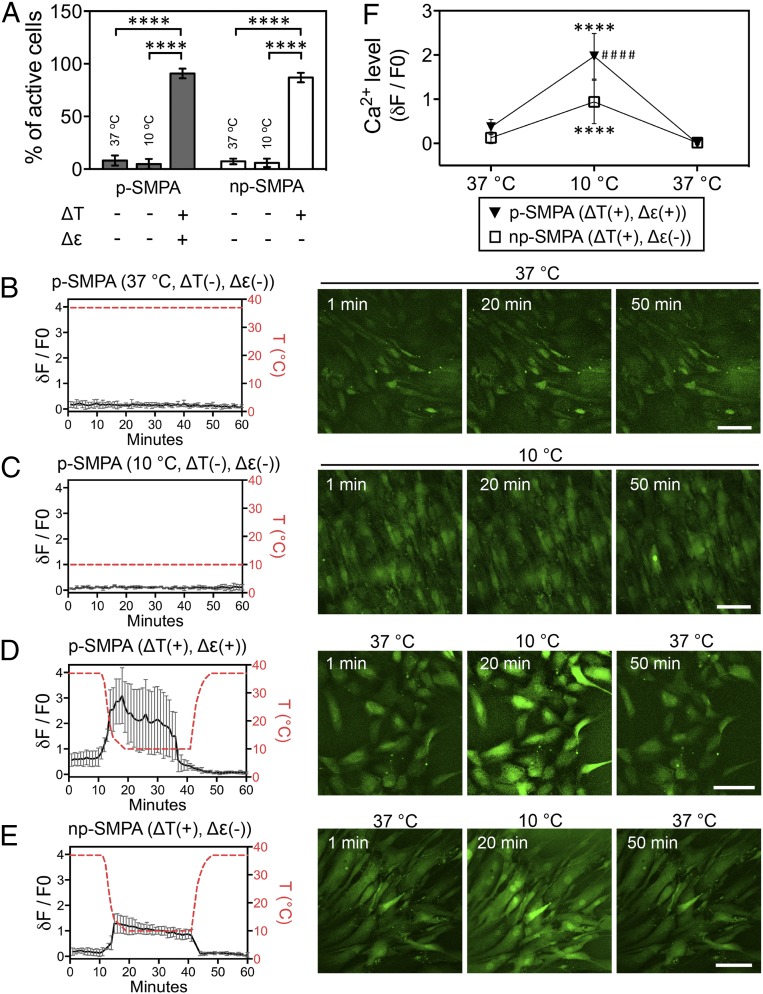
Ca^2+^ dynamics of hADSCs exposed to thermal and mechanical dual stimuli. (*A*) Percentage of cells with visible active Ca^2+^ influx in one temperature change cycle on p-SMPA and np-SMPA sheets. The cells were cultured in competitive differentiation medium and the intracellular Ca^2+^ was labeled with Fluo-4 calcium indicator. Quantification was based on ≥3 randomly selected SMPA sheets with more than 46 cells per group (****P < 0.0001, one-way ANOVA with Tukey’s multiple comparisons test). (*B* and *C*) Intracellular Ca^2+^ fluorescence in hADSCs without active Ca^2+^ influx on p-SMPA sheets exposed to a constant temperature of either 37 or 10 °C (*n* ≥ 3). (*D* and *E*) Intracellular Ca^2+^ fluorescence in hADSCs with active Ca^2+^ influx growing on p-SMPA (*n* = 7) and np-SMPA (*n* = 4) exposed to cyclic temperature change. The representative confocal images in *B*–*E* showed the fluorescence of intracellular Ca^2+^. (Scale bar, 100 µm.) (*F*) Intracellular Ca^2+^ of hADSCs in one temperature-changing cycle on p-SMPA (*n* = 50) and np-SMPA (*n* = 26) (****P < 0.0001, compared to corresponding values at 37 °C; ^####^*P* < 0.0001, p-SMPA vs. np-SMPA at 10 °C; one-way ANOVA with Tukey’s multiple comparisons test).

As a signaling messenger, Ca^2+^ plays a key role in the cellular signaling cascades that regulate the migration, proliferation, and differentiation of MSCs (25, 26). Here, the intracellular Ca^2+^ concentration is likely mediated by different Ca^2+^ influx/efflux pathways including entry into the cell membrane, release from intracellular stores such as the endoplasmic reticulum, and being pumped back from cytosol to the extracellular environment or into intracellular stores (27). Given that thermal and mechanical dual stimuli generated shape and orientation change in hADSCs, we investigated their effect on thermo- and mechanosensitive ion channels, and subsequent intracellular Ca^2+^ concentration. A significantly higher percentage of cells (87 ± 4.5% on np-SMPA and 90.9 ± 4.6% on p-SMPA) exposed to cyclic temperature change displayed visible Ca^2+^ influx (Fig. 2*A* and Movies S5 and S6). In contrast, only a small fraction of cells showed visible Ca^2+^ influx when cultured at 37 °C (7.3 ± 2.6% on np-SMPA and 8.1 ± 4.8% on p-SMPA) and at 10 °C (5.9 ± 4% on np-SMPA and 4.9 ± 4.7% on p-SMPA) (Fig. 2*A*), while the majority of cells displayed no visible Ca^2+^ influx (Fig. 2 *B* and *C* and *SI Appendix*, Fig. S6 *A* and *B*).

The analysis of the dynamic intracellular Ca^2+^ levels suggested a rapid variation Ca^2+^ concentration with temperature change (Fig. 2 *D*–*F*). Given that the properties of the SMPA, such as polymer crystallization and elasticity, are temperature dependent, these experiments were repeated on glass, with similar results observed (*SI Appendix*, Fig. S5*A* and Movie S7). The cyclic temperature change between 37 and 10 °C induced a greater Ca^2+^ influx and activation than with the temperature range 37 and 30 °C (*SI Appendix*, Fig. S5 *B*–*D*). This observation verified that certain thermosensitive channels were responsible for the temperature-induced Ca^2+^ influx. For example, the thermosensitive TRPM8 channel is activated below 20 °C, facilitating the intracellular entry of Ca^2+^ (16). The inhibition of Ca^2+^ entry and release using 2APB would effectively decrease the temperature-induced elevation of intracellular Ca^2+^ (*SI Appendix*, Fig. S5 *E* and *F*). Despite the actuation’s negligible influence on the percentage of cells which experienced Ca^2+^ influx, it contributed to the Ca^2+^ signaling by increasing the intracellular Ca^2+^ level. The cells exposed to dual stimuli presented a higher concentration of intracellular Ca^2+^ than those exposed to thermal stimulus (Fig. 2 *D*–*F* and *SI Appendix*, Fig. S6*C*). Compared to the control, a significantly higher intracellular Ca^2+^ level was observed in the group with only mechanical stimulus (*SI Appendix*, Fig. S6*C*). This confirmed that the actuation of the sheet might activate some mechanosensitive channels, such as TRPM7 (28) and PIEZO1 (29). Previous work has shown that the proliferation and differentiation of stem cells can be regulated by spontaneous Ca^2+^ oscillations (30, 31). Dynamic intracellular Ca^2+^ imaging showed that the spontaneous Ca^2+^ oscillations could be still observed (*SI Appendix*, Fig. S5*G*), indicating the preservation of spontaneous Ca^2+^ oscillations under the dual stimuli.

### The Role of Mechanical Stimuli and Intracellular Ca^2+^ on YAP Signaling Activation and Suppression.

When incident on cells, an external mechanical signal can regulate integrin activation, focal adhesion composition, and cytoskeleton organization to transduce the mechanical signal intracellularly and activate a series of downstream pathways. Talin, a vital component of integrin adhesion complex which mediates integrin activation and connects integrin to cytoskeleton (32), was enriched at the lamellipodia of hADSCs upon SMPA actuation (*SI Appendix*, Fig. S7*A*). Compared to the cells without stimuli and only thermal stimulus, the cells in the group of dual stimuli expressed increased integrin β1 as well as enhanced fibronectin fibrillogenesis (*SI Appendix*, Fig. S7 *B* and *C*), suggesting an effect of mechanical stimuli on matrix protein assembly and activation of integrin adhesion complexes.

**Fig. 3.**
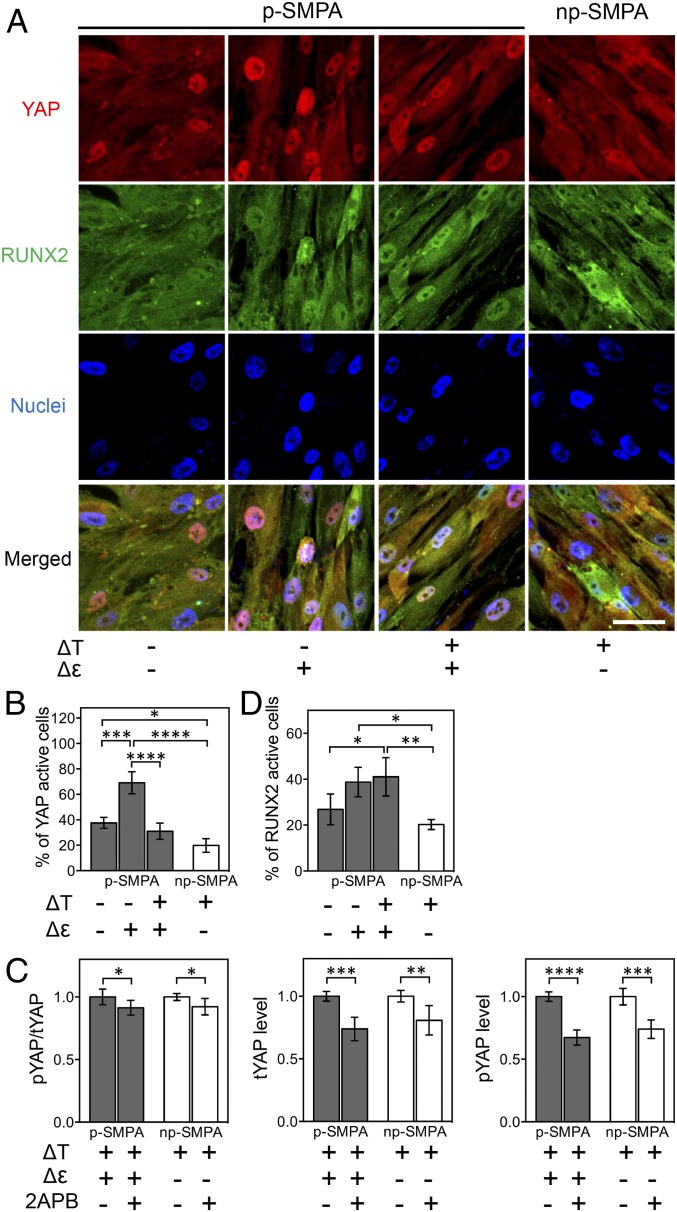
Regulation of YAP and RUNX2 nuclear translocation by thermal and mechanical stimuli. (*A*) Representative images of YAP and RUNX2 immunostaining. (Scale bar, 50 µm.) (*B*) Percentage of nuclear YAP positive cells (two-way ANOVA, *P* < 0.0001 for effect of ΔT, *P* < 0.001 for effect of Δε, *P* < 0.05 for ΔT × Δε interaction; *P < 0.05, ***P < 0.001, ****P < 0.0001, Tukey’s multiple comparisons test). (*C*) Ratio of phosphorylated YAP (pYAP) to total YAP (tYAP), levels of pYAP and tYAP of hADSCs cultured on SMPA sheets with and without Ca^2+^ inhibition. The enzyme-linked immunosorbent assay was based on lysate of cells on ≥5 independent SMPA sheets in each group, and the obtained value were normalized to the amount of total protein in the cell lysate (*P < 0.05, **P < 0.01, ***P < 0.001, ****P < 0.0001, Student’s *t* test). The value of the group without inhibition was set to 1 as a reference point. (*D*) Percentage of nuclear RUNX2 positive cells (two-way ANOVA, *P* < 0.001 for effect of Δε, nonsignificant for effect of ΔT and ΔT x Δε interaction; *P < 0.05, **P < 0.01, Tukey’s multiple comparisons test). For *B* and *D*, ≥3 randomly selected images containing more than 200 cells in each group were analyzed. The cells were cultured in competitive differentiation medium for 10 d.

It has been suggested that YAP and TAZ are sensors for mechanical signals and act as an intracellular rheostat, storing past mechanical information and regulating differentiation of stem cells. Particularly, YAP/TAZ play a key role in mediating the translation of incoming mechanical information into the expression of osteogenesis-related transcriptional factors (9). The shape change of the p-SMPA sheet significantly enhanced YAP and RUNX2 activity (nuclear localization) (Fig. 3 *A*, *B*, and *D* and *SI Appendix*, Fig. S8). Inhibiting of actin polymerization with cytochalasin D (CytoD) to block the mechanotransduction pathway resulted in significantly decreased YAP and RUNX2 activity (*SI Appendix*, Fig. S9 *B* and *D*). However, the nuclear localization of YAP was suppressed by the cyclic temperature change (Fig. 3*B*). The elevation of cytosolic Ca^2+^ could inhibit YAP/TAZ activity through the Hippo pathway, phosphorylating YAP/TAZ and preventing their nuclear accumulation (33). Therefore, the decreased YAP activity could be attributed to the enhanced intracellular Ca^2+^ concentration during temperature change. Inhibition of Ca^2+^ influx with 2APB suppressed YAP phosphorylation and the expression of total YAP (Fig. 3*C*), but increased the percentage of nuclear YAP active cells (*SI Appendix*, Fig. S9 *A* and *C*), which confirmed the role of Ca^2+^ in regulating YAP activity. The SMPA sheet’s geometric and temperature changes could regulate RUNX2 activity through a signaling network consisting of multiple pathways (see summary in Fig. 6). Either mechanical stress could activate RUNX2 through the activation of YAP, or an increase of intracellular Ca^2+^ concentration, induced by stretch and temperature change, might exert both a negative and positive regulatory effect on RUNX2 activity. For example, Ca^2+^ could inhibit RUNX2 through deactivation of YAP but activate RUNX2 via Ras/ERK1/2, MAPK, and CaMKII signaling (34–36). Here, the nuclear localization of RUNX2 was influenced by the SPMA sheet deformation but not by temperature change (Fig. 3*D*).

### Histone Modification Induced by Thermal and Mechanical Stimuli.

**Fig. 4.**
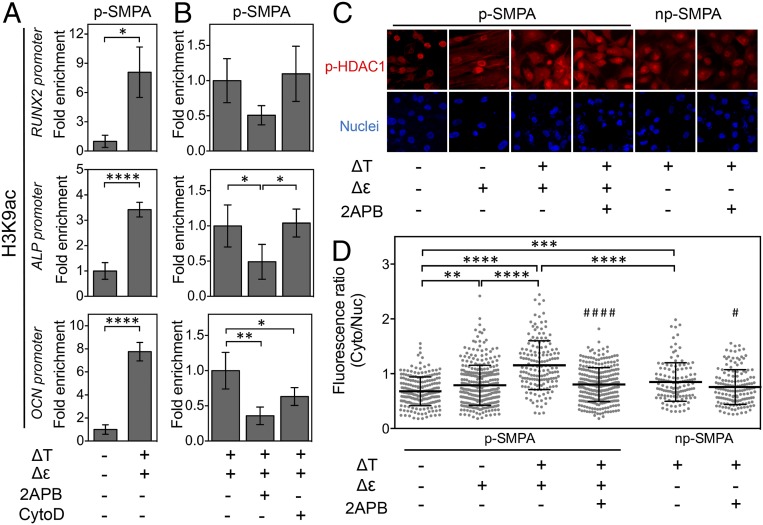
Epigenetic alternation of hADSCs exposed to thermal and mechanical stimuli. (*A*) H3K9 acetylation level at the promoters of *RUNX2, ALP,* and *OCN* genes on p-SMPA sheet without and with cyclic temperature change. The values of the group without temperature change were set as 1 (*n* ≥ 4; *P < 0.05, ****P < 0.0001, Student’s *t* test). (*B*) Regulation of Ca^2+^ signaling and mechanotransduction on H3K9 acetylation in hADSCs perceiving dual stimuli. Cells were treated with Ca^2+^ inhibitor (2APB) and actin inhibitor (CytoD). The values of the group without inhibition were set as 1 (*n* = 4; *P < 0.05, **P < 0.01, one-way ANOVA with Tukey’s multiple comparisons test). (*C*) Staining of p-HDAC1 (red) and nuclei (blue) of hADSCs cultured in different conditions. (Scale bar, 20 µm.) (*D*) Ratio of fluorescence intensity of the cytoplasm and nuclear p-HDAC1 on SMPA sheets. This quantification was based on the images of more than 126 cells from 3 randomly selected SMPA sheets in each group [two-way ANOVA, *P* < 0.0001 for effects of ΔT and Δε, *P* < 0.001 for ΔT × Δε interaction in the absence of 2APB; **P < 0.01, ***P < 0.001, ****P < 0.0001, Tukey’s multiple comparisons test; ^#^*P* < 0.05, ^####^*P* < 0.0001, 2APB(+) vs. 2APB(−) with the same stimuli, Student’s *t* test]. The cells were examined after being cultured in competitive differentiation medium for 10 d.

To gain insight into the effect of the dual stimuli at the epigenetic level, histone modification was evaluated after 10 d. The H3K9 acetylation (H3K9ac) at promoters of osteogenesis-related genes, including *RUNX2,* alkaline phosphatase (*ALP*), and osteocalcin (*OCN*), was significantly enhanced in the cells treated with dual stimuli (Fig. 4*A*). Inhibition of intracellular Ca^2+^ with 2APB significantly decreased the level of H3K9ac at *ALP* and *OCN* promoters. Treatment with CytoD, which inhibits actin, decreased the H3K9ac at *OCN* promoter but not at *RUNX2* and *ALP* promoters (Fig. 4*B*). The effect of thermal or mechanical stimulus on histone modification was also observed in H3K27 trimethylation (H3K27me3). Treatment with 2APB increased the H3K27me3 at the promoters of *RUNX2* and *ALP,* and inhibition of actin led to the elevation of H3K27me3 at *ALP* promoter. However, neither 2APB nor CytoD treatment showed effect on H3K27me3 at *OCN* promoter (*SI Appendix*, Fig. S10). These results suggested that both Ca^2+^ and mechanical signals could modulate histone modification, with different role for different types of histone modification.

The histone deacetylase 1 (HDAC1) is an important regulator of histone acetylation. Phosphorylation of HDAC1 at Ser421 increases its deacetylation activity. Previous studies have demonstrated the response of HDAC1 expression, nuclear localization, and phosphorylation to external mechanical cues and Ca^2+^ signaling. For example, HDAC1 expression was down-regulated by cyclic stretch (37). Mechanical stimuli of interstitial flow stimulated HDAC1 phosphorylation and increased the localization of phosphorylated HDAC1 (p-HDAC1) to the cytoplasm (38). Intracellular Ca^2+^ inhibited nuclear localization and phosphorylation of HDAC1 (39, 40). Here, the subcellular localization of p-HDAC1 was investigated by analyzing the fluorescence intensity of immunostained p-HDAC1 in cytosol and nuclei (Fig. 4 *C* and *D*). Both thermal and mechanical changes stimulated the nuclear export of p-HDAC1 and when synchronized generated the highest level of cytosolic localization of p-HDAC1. Inhibition of Ca^2+^ eliminated the increased nuclear export of p-HDAC1, suggesting that Ca^2+^ plays a key role in regulating the subcellular distribution of p-HDAC1. Therefore, the potential mechanism for enhanced H3K9ac at the promotors of osteogenic genes can be attributed to the regulation of dual stimuli on histone deacetylase.

### Promotion of Osteogenesis and Suppression of Adipogenesis of hADSCs by Thermal and Mechanical Stimuli.

**Fig. 5.**
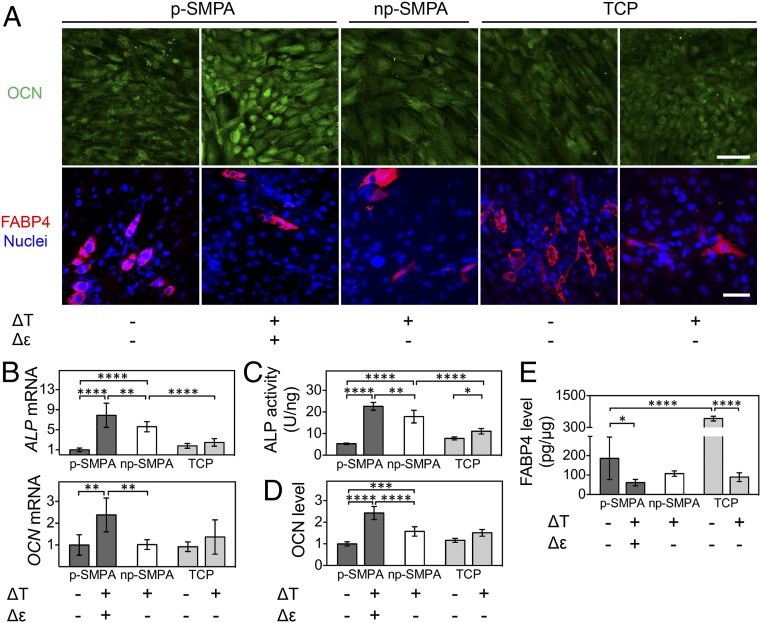
The effect of thermal and mechanical dual stimuli on hADSC differentiation. (*A*) OCN (green) and FABP4 (red) immunostaining on SMPA sheets and TCP under different conditions. (Scale bar, 100 µm.) (*B*) Expression levels of *ALP* and *OCN* mRNA (*n* ≥ 5). The values of the group on SMPA without thermal and mechanical stimuli were set as 1. (*C*) ALP activity of hADSCs cultured under different conditions (*n* ≥ 3). (*D*) OCN fluorescence intensity normalized to cell number. The analysis was based on five randomly selected images in each group. (*E*) The enzyme-linked immunosorbent assay of FABP4 of hADSCs (*n* ≥ 4). To induce cell differentiation, the competitive differentiation medium was applied for 3 wk. (*P < 0.05, **P < 0.01, ***P < 0.001, ****P < 0.0001, one-way ANOVA with Tukey’s multiple comparisons test.)

To investigate the effect of dual stimuli on hADSCs’ fate decision, differentiation markers were quantified at mRNA and protein levels (Fig. 5). Both temperature change and p-SMPA actuation promoted the osteogenesis of hADSCs. Although no dramatic influence on OCN was observed, temperature change significantly increased *ALP* mRNA and protein activity (Fig. 5 *A*–*C*). The actuation of p-SMPA sheet enhanced the gene expression of *ALP* and *OCN*, as well as the protein level of OCN and ALP activity (Fig. 5 *A*–*D*), and increased the cellular mineralization of differentiated hADSCs (*SI Appendix*, Fig. S11*A*). The OCN level under dual stimuli was significantly higher than that under mechanical stretch only (*SI Appendix*, Fig. S11 *B* and *C*), suggesting a cumulative effect of temperature change and p-SMPA actuation on osteogenesis. The adipogenesis of hADSCs was shown to be inhibited by temperature change and p-SMPA actuation. The adipogenesis marker FABP4 was down-regulated in samples exposed to cyclic temperature change and was further decreased in the presence of both stimuli (Fig. 5 *A* and *E*). The amount and size of lipid droplets identified via ORO staining significantly decreased in cells exposed to cyclic temperature change compared to those held at 37 °C (*SI Appendix*, Fig. S11 *D* and *E*).

**Fig. 6.**
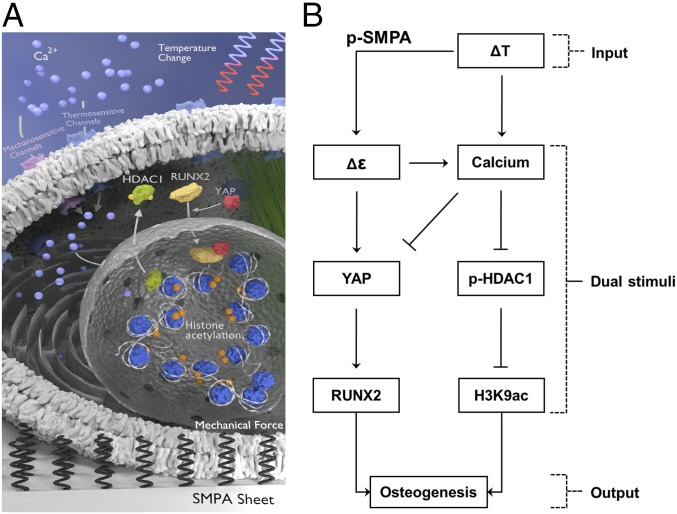
The influence of thermal and mechanical dual stimuli on hADSCs. (*A*) A schematic illustration showing the effect of temperature change and the actuation of the p-SMPA on Ca^2+^ signaling via thermo- and mechanosensitive channels as well as on YAP activity via mechanotransduction. Ca^2+^ signaling enhanced H3K9 acetylation while YAP activated RUNX2, both of which promoted osteogenesis of hADSCs. (*B*) Flowchart illustrating the function of p-SMPA as a signal processer to convert an input (temperature change) into dual stimuli, subsequently regulating the differentiation of hADSCs as an output.

In summary, a thermally controlled SMPA sheet was employed to control the differentiation process of MSCs. In this work, we demonstrate that multiple physical stimuli (physical force, temperature etc.) provided by a programmed polymeric actuator sheet could be used as basic inputs to guide the construction of the internal signal processing architecture of living cells (Fig. 6). The results of our study suggest that the thermo- and mechanosensing networks of stem cells are interconnected via a universal intracellular component. The actuation of a sheet during cyclic temperature change imparts a mechanical and cold stress on hADSCs, which are logically connected to their discrete networks by Ca^2+^. Ca^2+^ dynamics regulate the intercellular connection of mechano- and thermosensing components, serving as a cellular basis for the osteogenic differentiation of MSCs. Polymer actuation activates the mechanosensor YAP, and subsequently promotes RUNX2 nuclear localization. Meanwhile, the cyclic temperature change and interlinked polymer sheet actuation decreased nuclear localization of phosphorylated HDAC1 via Ca^2+^ signaling, and maintained a high level of histone H3K9 acetylation on osteogenesis-related gene promoters. Thermal and mechanical dual effects work cooperatively to promote osteogenic differentiation of hADSCs. This dual-stimuli system could serve as a culture device to modulate stem-cell function, demonstrating the cooperative use of multiple external signals to achieve biological outputs. The p-SMPA sheet shows great potential to meet the clinical requirements for the tissue engineering of periosteum to treat bone defects. MSCs could be cultured in vitro where both thermal and mechanical stimuli are used to induce a high level of osteogenesis. After transplantation, the shape-memory effect of a p-SMPA sheet could allow self-tightening when exposed to the relatively higher temperature of the body, facilitating the attachment of the cell-laden sheet onto bone and accelerating its restoration process. In the future, detailed knowledge of signaling mechanisms with both spatial and temporal resolution is required. It is important to use a systemic approach, logically dissect the thermal and mechanical stimuli, and establish a precise and faithful model system, which enables a more effective combination of different stimuli.

## Materials and Methods

Detailed descriptions appear in *SI Appendix*.

### Generation of Thermal and Mechanical Stimuli and Cyclic Temperature Change.

The cyclic temperature change between 37 and 10 °C was realized by computer-controlled thermochambers (Instec). Thermal and mechanical dual stimuli were generated by subjecting p-SMPA to temperature cycles. In the case of np-SMPA sheet only a thermal stimulus is provided, and a mechanical stretching device was used to generate mechanical stimulation.

### In Situ Measurement of Temperature, SMPA Sheet Shape, and Cell Morphology.

Temperature and cell morphology were measured using a digital thermometer and laser scanning microscopy, respectively, where the 50 × 50-µm grid printed on the SMPA sheet was used to provide information about the actuation of the cell substrate during characterization.

### Data Availability.

The raw data for Figs. 1–5 are available at DOI: 10.6084/m9.figshare.c.4682696.v1.

## Supplementary Material

Supplementary File

Supplementary File

Supplementary File

Supplementary File

Supplementary File

Supplementary File

Supplementary File

Supplementary File
