# Supplementary material for: Polymeric sheet actuators with programmable bioinstructivity
Source: Proc Natl Acad Sci U S A. 2020 Jan 13;117(4):1895–901. doi: 10.1073/pnas.1910668117 (PMC6995006; doi:10.1073/pnas.1910668117)
Supplement: Supplementary File [file pnas.1910668117.sapp.pdf]

PNAS
www.pnas.org

Supplementary Information for

Polymeric Sheet Actuators with Programmable Bioinstructivity

Zijun Deng[#], Weiwei Wang[#], Xun Xu[#], Oliver E. C. Gould, Karl Kratz, Nan Ma and Andreas Lendlein
# These authors contributed equally to this work.

Nan Ma, Andreas Lendlein
Email: nan.ma@hzg.de, andreas.lendlein@hzg.de

This PDF file includes:

Supplementary text
Figures S1 to S12
Legends for Movies S1 to S7
SI References

Other supplementary materials for this manuscript include the following:

Movies S1 to S7

www.pnas.org/cgi/doi/10.1073/pnas.1910668117

Supplementary Information Text

Supplementary Materials and Methods.

SMPA Preparation. Linear poly(ε-caprolactone) - diisocyanatoethyl dimethacrylate (PCL-DIEMA) was synthesized according to a previously reported free radical polymerization method (1). 2,2'-Azobisisobutyronitrile (AIBN), chloroform, and inhibitor remover were purchased from Sigma Aldrich. AIBN was used after crystallization from ethanol. N-butyl acrylate (Merck) was passed over inhibitor remover before use. Hexane was purchased from Acros and used as received. Diisocyanatoethyl dimethacrylate was synthesized according to a previously reported method (2).

Polymers of Mn of 3,000 g.mol-1 and 8,000 g.mol-1 were mixed in a weight ratio of 1:5 and ground into a homogenous mixture according to (2). 40% wt of this mixture to 60% wt of n-butyl acrylate was heated to 50 °C for 15 minutes before the initiator AIBN (1 wt%) was added whilst stirring carefully to prevent air bubbles forming. After 3 min, stirring was stopped. The obtained mixture was poured into a mold consisting of a glass plate patterned with 50 x 50 µm grids, a flat glass plate and a spacer of 0.5 mm in thickness, then kept in an oven at 80 °C overnight. The sheet was cut into strips, washed with chloroform twice, then once with a 50:50 mixture of chloroform: hexane, and then dried under vacuum at 70 °C for 7 days. Programming of the polymer sheet was performed by stretching to ε_{prog} of 60% at T_{prog} = 50 °C before cooling to - 20°C under constant strain. Finally, the polymer was punched into circular sheets with a diameter of 10 mm, heated to 40 °C and left for 10 minutes.

Generation of Thermal and Mechanical Stimuli and Cyclic Temperature Change. The cyclic temperature change between 37 °C and 10 °C was realized by computer controlled thermochambers (Instec, Colordao, USA) suitable for a standard 35 mm cell culture dish (smaller chamber) on an inverted microscope or two standard 24-well tissue culture plates (larger chamber). The 35 mm dish and each well of the 24-well plates were filled with 1.5 mL and 0.6 mL liquid (PBS or cell culture medium), which covered the polymer sheet, and supplied with 5 vol% CO_2 for cell maintenance. For each cycle of temperature change, the time was set to 60 minutes, consisting of 8 minutes to cool from 37 °C to 10 °C, 22 minutes at 37 °C, 8 minutes to heat from 10 °C to 37 °C, and 22 minutes at 10 °C.

Thermal and mechanical dual stimuli were generated by subjecting p-SMPA to temperature cycles. In the case of non-programmed SMPA sheet only a thermal stimulus was provided. A stretching device that enables the cyclic stretch deformation of the SMPA sheet at 37 °C was used to produce to expose the cells to the mechanical stimulus. SMPA at constant temperature (37 °C) exhibited neither thermal nor mechanical stimulus.

Mechanical Stimulation at Constant Temperature A stretching device (MCB1, CellScale, Ontario, Canada) was used to generate the cyclic stretch of the SMPA sheet at a constant temperature of 37 °C. The device was put into a cell culture incubator (37 °C, 5 vol% CO2). The programmed SMPA sheets were fixed and stretched along the

direction of programming (Fig. S12). In order to have the same shape change behavior as inside the thermal chamber, the time was set to 60 minutes for each stretching cycle, consisting of 8 minutes of stretching, 22 minutes to hold, 8 minutes to release, 22 minutes to hold. The SMPA sheet was stretched to 10% elongation in each cycle to match the cyclic temperature change experiments.

hADSC Culture and Differentiation The hADSCs were isolated from human adipose tissue after informed consent (No.: EA2/127/07; Ethics Committee of the Charité – Universitätsmedizin Berlin, approval from 17.10.2008), as described in a previous study (3). The cells were held at a constant temperature of 37 °C in an incubator supplied with 5 vol% CO_2, in cell growth medium - DMEM medium (Thermo Fisher Scientific, Schwerte, Germany) suppled with 10 vol% fetal bovine serum (Sigma Aldrich, Hamburg, Germany) and 1 vol% non-essential amino acids (Thermo Fisher Scientific, Schwerte, Germany). The medium was changed every 2 days and the cells were passaged at a ratio of 1:3 when the confluence reached ~90%.

In order to examine the cell differentiation on SMPA sheets, the competitive differentiation medium consisting of 1:1 (v:v) adipogenesis medium (StemPro® adipogenesis differentiation kit, Thermo Fisher Scientific, Schwerte, Germany) and osteogenesis medium (StemPro® osteogenesis differentiation kit, Thermo Fisher Scientific, Schwerte, Germany) were used. The medium was changed every 3 days.

In-Situ Measurement of SMPA Sheet Shape and Cell Morphology

The temperature of the liquid surrounding the SMPA sheet was measured using a digital thermometer. The SMPA sheets and the cells were monitored using laser scanning confocal microscopy (LSM 780, Carl Zeiss, Jena, Germany) or a fluorescence microscope (Axiovert 200M, Carl Zeiss, Jena, Germany).

The 50 x 50 µm grid squares created on the bottom of SMPA sheet were measured in the direction of elongation and compression to analyze the shape change of SMPA sheets (ImageJ software, National Institutes of Health). The movement speed of the p-SMPA sheet during actuation was calculated according to the measurements of dynamic shape change using a benchmark point 100 µm away.

The shape change of the cells growing upon p-SMPA sheet actuation was quantified by measuring the cell dimension in the direction of p-SMPA sheet elongation and compression as well as the cell surface area. The cell membrane, cytoplasm and nuclei were stained using a CellMask™ Deep Red plasma membrane staining kit, CFSE and Hoechst 33342 respectively (Thermo Fisher Scientific, Schwerte, Germany). The orientation of cells on SMPA sheets was analyzed by measuring the angle between the direction of p-SMPA sheet elongation and the long axis of cells. In order to study the focal adhesion dynamics of cells upon p-SMPA actuation, the cells were transfected using CellLight® Talin-GFP, BacMam 2.0 (Thermo Fisher Scientific, Schwerte, Germany) to label talin with (GFP).

The GFP signal of cells cultured on p-SMPA sheets during cyclic temperature change was detected using laser scanning confocal microscopy.

Cell viability assay Live/dead staining of hADSCs was performed using CFSE and propidium iodide (PI) (Thermo Fisher Scientific, Schwerte, Germany). In brief, 25 μM CFSE was loaded to cells before cell seeding. At indicated time points, PI (2 μg/mL) was directly added to cell culture medium for visualizing dead cells. Images were taken by confocal laser scanning microscope (LSM780, Carl Zeiss, Jena, Germany) and analyzed using ImageJ software. CellTiter-Glo Luminescent cell viability assay kit and MultiTox-Fluor Multiplex cytotoxicity assay kit (Promega GmbH, Mannheim, Germany) were used to quantify the viable and dead cells simultaneously. At indicated time points, the conditioned medium was collected and the cell lysate was harvested immediately to determine the relative number of dead and viable cells, respectively. The cell viability was presented as the percentage of viable cell in total cell number (viable+dead cells).

Cell proliferation assay The hADSCs were seeded at a density of 0.25 x $10^4/cm^2$ and cultured in growth medium. After 3 days, the cell proliferation activity was assessed by staining the cell proliferation marker Ki67. In brief, 1 x $10^4/cm^2$ cells (in growth medium) were seeded for image based cell counting. To test the cell proliferation during differentiation, the hADSCs were seeded at a density of 2.5 x $10^4/cm^2$ and cultured in competitive differentiation medium. The cell number was counted based on the carboxyfluorescein succinimidyl ester (CFSE) and nuclear labeling (Hoechest 33342) (Thermo Fisher Scientific, Schwerte, Germany) or examined using a cell counting kit-8 (CCK-8, Dojindo, Offenbach, Germany).

Calcium Imaging. Intracellular Ca^{2+} was measured using a Fluo-4 kit according to the manufacturer's instructions. Real-time fluorescence images were acquired at 1 min intervals using laser scanning confocal microscopy and analyzed using ImageJ software. The fluorescence ratio δF/F0 (δF = Ft-F0) was calculated according to existing literature (40) for each cell, where Ft represents the measured fluorescence intensity and F0 corresponds to the lowest fluorescence level in each cell.

Intracellular calcium quantification. The intracellular calcium level of hADSCs was detected using a calcium quantification assay kit-Red fluorescence (Abcam, Berlin, Germany) according to the product manual. Cells cultured under different conditions were lysed using RIPA buffer (Thermo Fisher Scientific, Schwerte, Germany). Rhod Red calcium indicator was then added to the cell lysis and incubated for 30 min at room temperature. The fluorescence were measured by Tecan Infinite® 200 PRO microplate reader (Tecan Deutschland GmbH, Germany). The results were normalized with the amount of total protein for each sample.

Chemical Inhibition. The 2-aminoetho-xydiphenylborate (2APB, Sigma Aldrich, Hamburg, Germany) was used to block the Ca^{2+} entry through TRP channels as well as calcium release from endoplasmic reticulum by inhibiting

Inositol 1,4,5-trisphosphate receptors (InsP3Rs). For the calcium imaging experiments, 2APB was added to the medium 30 min before the experiment to reach a final concentration of 200 μM. For long-term experiments (10 days) into Ca^{2+} inhibition, 100 μM 2APB was applied and refreshed in every 3 days after medium change. Cytochalasin D (CytoD, Sigma Aldrich, Hamburg, Germany) was used to inhibit actin dynamics. The medium containing 0.5 μM CytoD was used to culture hADSCs for 10 days. For short-term inhibition experiments, the cells were incubated with 100 μM 2APB and 0.5 μM CytoD for 3 hours.

Cell Staining. For immunostaining, cells were fixed with 4 wt% paraformaldehyde (Sigma Aldrich, Hamburg, Germany), permeabilized using 0.25 vol% Triton X-100 (Sigma Aldrich, Hamburg, Germany), and blocked using 5 vol% goat serum (Thermo Fisher Scientific, Schwerte, Germany) in PBS. Samples were incubated with primary antibodies overnight at 4 °C, followed by incubation with the secondary antibodies (Thermo Fisher Scientific, Schwerte, Germany) for 1 hour at room temperature. The following primary antibodies were used: Ki67 (rabbit monoclonal, Cell Signaling Technology, Frankfurt am Main, Germany), YAP (rabbit monoclonal, Cell Signaling Technology, Frankfurt am Main, Germany), RUNX2 (mouse monoclonal, Abcam, Berlin, Germany), FABP4 (rabbit polyclonal, Thermo Fisher Scientific, Schwerte, Germany), Osteocalcin (mouse monoclonal, Cell Signaling Technology, Frankfurt am Main, Germany), phosphor-HDAC1 (rabbit polyclonal, Thermo Fisher Scientific, Schwerte, Germany), fibronectin (rabbit polyclonal, Abcam, Berlin, Germany) and integrin β1 (mouse monoclonal, R&D Systems, Minneapolis, USA). YAP and RUNX2 nuclear localization was analyzed by comparing fluorescence intensity in the nucleus and cytoplasm of each cell using ImageJ (National Institutes of Health). A cell with nuclear/cytoplasm fluorescence ratio > 1.35 was defined as a YAP or RUNX2 active cell. The cell nuclei were stained with 4',6-diamidino-2-phenylindole (DAPI) for 5 minutes at room temperature. A confocal laser scanning microscope (LSM780, Carl Zeiss, Jena, Germany) was used to observe the stained cells.

Oil Red O staining was performed to visualize the lipids of cells undergoing adipogenesis. After 2 weeks of adipogenic induction, the cells were washed with PBS, fixed in 4 wt% paraformaldehyde (Sigma Aldrich, Hamburg, Germany) and stained using 0.1 wt% Oil Red O (Sigma Aldrich, Hamburg, Germany) in isopropanol for 15 minutes. After washing 3 times with water, the Oil Red O was extracted using isopropanol. The absorbance at 490 nm of the mixture was measured using a microplate reader (Infinite 200 Pro, Tecan Group Ltd. Männedorf, Switzerland) to evaluate the efficiency of adipogenesis.

Alizarin Red S (ARS) was used to detect the calcium deposition of hADSCs during osteogenesis. Samples were washed with PBS and fixed in 4 wt% paraformaldehyde (Sigma Aldrich, Hamburg, Germany) at room temperature for 15 minutes. Then, 0.3 ml of 40 mM ARS (Sigma Aldrich, Hamburg, Germany) in water was added to each well. After 20 min incubation at room temperature, the samples were washed for 6 times with water.

Real time-PCR and ChIP-PCR The mRNA expression level of *ALP* and *OCN* was quantified with real time-PCR, using *GAPDH* as house-keeping gene. The total RNA was isolated using a mirVana Kit (Thermo Fisher Scientific, Schwerte, Germany), and quantified with a microplate reader (Infinite 200 Pro, Tecan Group Ltd. Männedorf, Switzerland) equipped with a NanoQuant Plate. The cDNA was synthesized using a RT^2 First Strand kit (Qiagen, Hilden, Germany). Then, the cDNA was mixed with RT^2 SYBR Green ROX qPCR Mastermix (Qiagen, Hilden, Germany) and corresponding primers (*ALP* 5'-CCCCCGTGGCAACTCTATCT-3' and , 5'-GATGGCAGTGAAGGGCTTCTT-3'; *OCN* 5'-AGCAAAGGTGCAGCCTTTGT-3' and 5'-GCGCCTGGGTCTCTTCACT-3'; *GAPDH* 5'-ATGGGGAAGGTGAAGGTCG-3' and 5'-GGGGTCATTGATGGCAACAATA-3'), and amplified using a StepOnePlus real-time PCR System (Thermo Fisher Scientific, Schwerte, Germany).

The chromatin immunoprecipitation (ChIP) was performed using a High-sensitivity ChIP kit (Abcam, Berlin, Germany) pre-coated with H3K9ac or H3K27me3 antibody (rabbit monoclonal, Cell Signaling Technology, Frankfurt am Main, Germany). The real-time PCR was applied to quantify the precipitated DNA using the primers targeting the promoter region of *RUNX2*, *ALP* and *OCN* (*RUNX2* 5'-AGGCCTTACCACAAGCCTTT-3' and 5'-AGAAAGTTTGCACCGCACTT-3'; *ALP* 5'-TCCAGGGATAAAGCAGGTC-3' and 5'-TTAGTAAGGCAGGTGCCAAT-3'; *OCN* 5'-CAAATAGCCCTGGCAGATTC-3' and 5'-GAGGGCTCTCATGGTGTCTC-3').

ELISA and ALP Activity Assay The cells were lysed using a cell lysis buffer. The FABP-4, phosphorylated YAP (pYAP) and total YAP (tYAP) in the cell lysate was quantified using the FABP4 ELISA kit (R&D Systems, Minneapolis, USA), PathScan® Phospho-YAP (Ser127) ELISA kit and PathScan® Total YAP ELISA kit (both were from Cell Signaling Technology, Frankfurt am Main, Germany) respectively. The ALP activity was measured using an alkaline phosphatase assay kit (Abcam, Berlin, Germany). The results were normalized with the amount of total protein for each sample, which was determined using a BCA protein assay kit (Thermo Fisher Scientific, Schwerte, Germany).

Statistical Analysis The number of repetitions for each experiment was indicated in the figure legends for each assay, with all data expressed as mean value ± standard deviation. Statistical analysis was performed using GraphPad Prism (Version 7.04, GraphPad Software, Inc., San Diego, CA, USA). The significance of the difference between two groups was determined using a two-tailed, unpaired Student's *t*-test. Differences among three or more independent groups were analyzed using one-way or two-way ANOVA followed by Tukey's multiple comparisons test. A p value less than 0.05 was considered to be statistically significant.

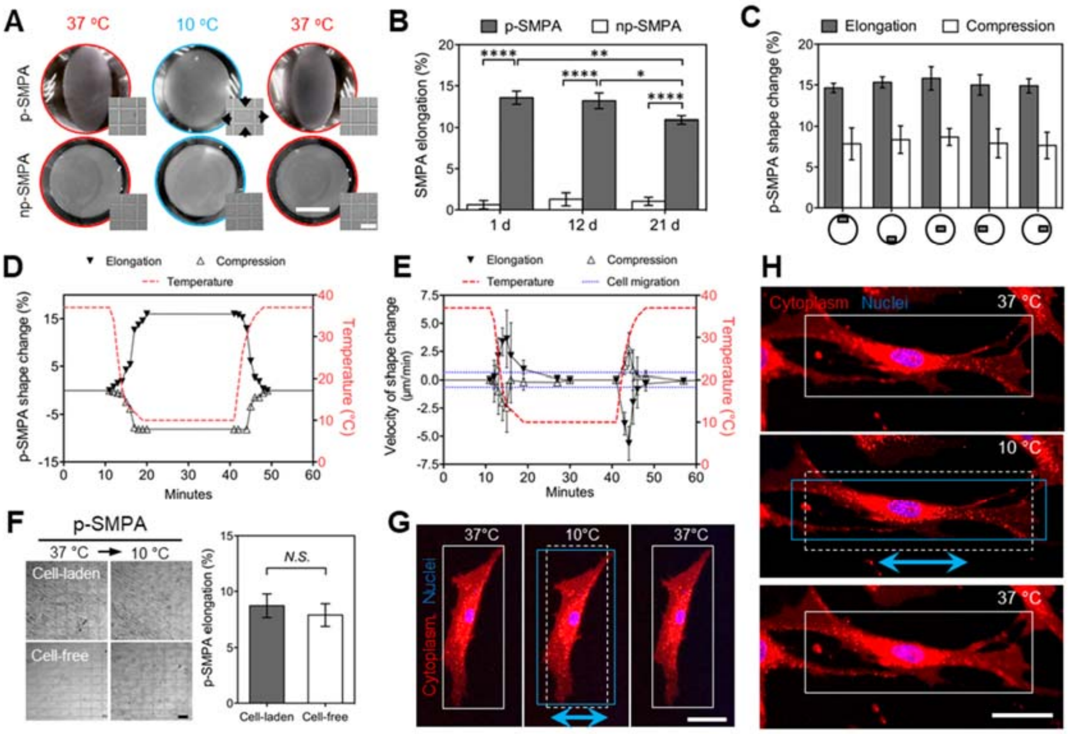

Fig. S1. (A) Representative images showing the macroscale shape change of non-programmed (np) and programmed (p) SMPA sheets in one cycle of temperature change in PBS solution. The elongation and compression of p-SMPA were analyzed according to the shape change of grid squares. Arrows indicate the elongation and compression directions (scale bar = 5 mm for SMPA sheets, scale bar = 50 μm for grid squares). (B) Quantification of p-SMPA shape change in 3 weeks in cell culture medium (n = 3; **p* < 0.05, ***p* < 0.01, *****p* < 0.0001, two-way ANOVA with Tukey's multiple comparisons test). (C) Temperature induced shape change of p-SMPA sheet in the center and near the edges of the p-SMPA sheet (n = 6). (D) Elongation and compression of p-SMPA sheet under the cyclic temperature change. (E) Shape change velocity of the p-SMPA sheet during actuation using a point of benchmark 100 μm away (n = 10). (F) Shape change of cell-laden and cell-free p-SMPA sheets under cyclic temperature change. Cells were cultured in growth medium on the p-SMPA sheet for 3 days (scale bar = 50 μm; n >= 12; N.S. non-significant, Student's *t*-test). (G, H) Fluorescent microscopy images of a single cell cultured in growth medium on p-SMPA sheet with temperature induced shape change. The blue arrows represent the direction of elongation. The solid white and blue boxes were used for positioning the cell border at 37 °C and 10 °C respectively (scale bar = 50 μm).

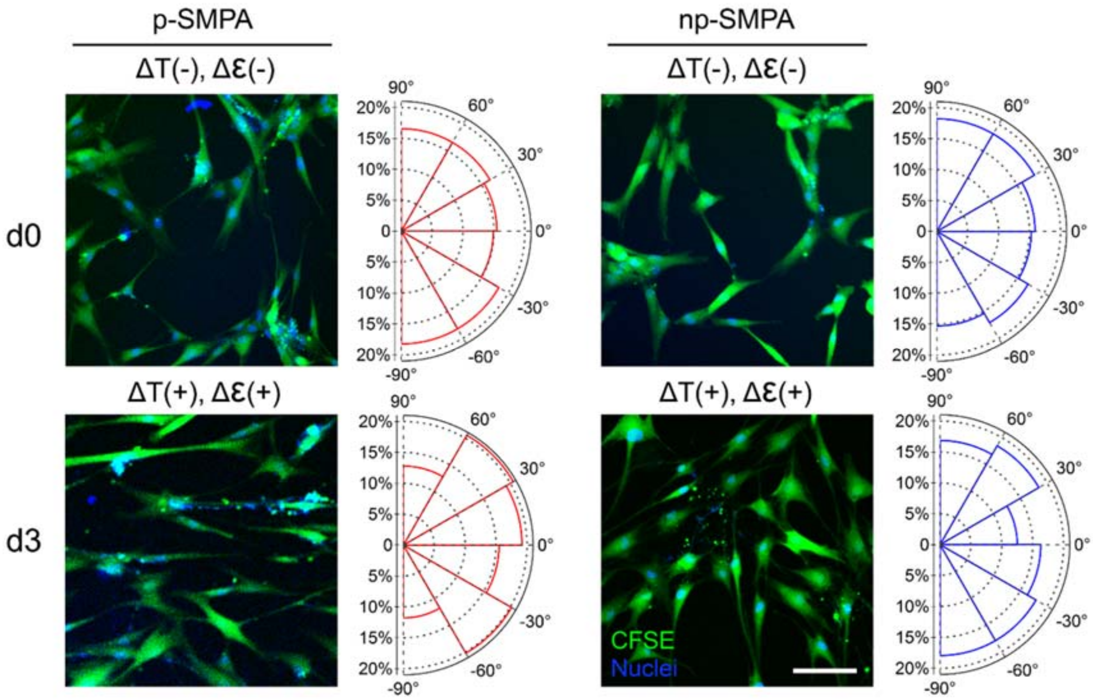

Fig. S2. Cell orientation at day 0 and after 3 days of cyclic temperature change on non-programmed (np) and programmed (p) SMPA sheets. Cells were cultured in competitive differentiation medium, and the living cells were stained with CFSE (green) and Hoechst 33342 (blue) to visualize cytoplasm and nuclei. For each group, more than 170 cells were analyzed to calculate the percentage of cells at different directions (scale bars = 100 μm).

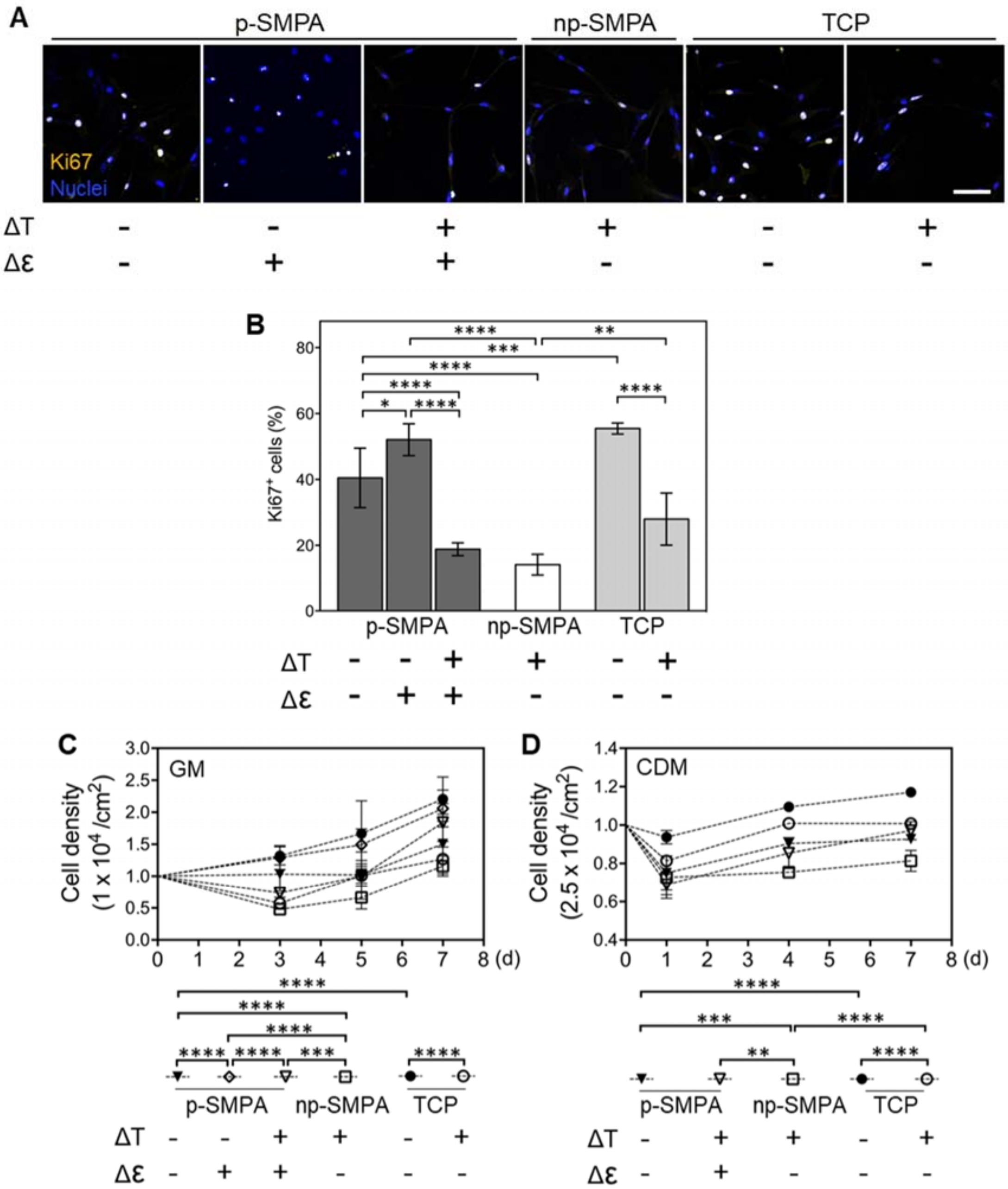

Fig. S3 (A) Ki67 staining (yellow) of cells cultured in growth medium for 3 days at different conditions. Cell nuclei were stained with DAPI (blue) (scale bar = 100 μm). (B) Percentage of Ki67 positive cells in each group. The quantification was based on 6 randomly selected images (*$p < 0.05$, **$p < 0.01$, ***$p < 0.001$, ****$p < 0.0001$, one-way ANOVA with Tukey's multiple comparisons test). (C) Cell proliferation on TCP and SMPA sheets in growth

medium under different stimuli. The cells were stained with CFSE and the images were taken at indicated time points. The cell number were determined by counting the cells on at least 5 randomly selected images using ImageJ software. (D) Cell proliferation on TCP and SMPA sheets in competitive differentiation medium under different stimuli. The cell number were quantified using a cell counting kit (n = 4). (**$p < 0.01$, ***$p < 0.001$, ****$p < 0.0001$ in C and D, two-way ANOVA with Tukey's multiple comparisons test)

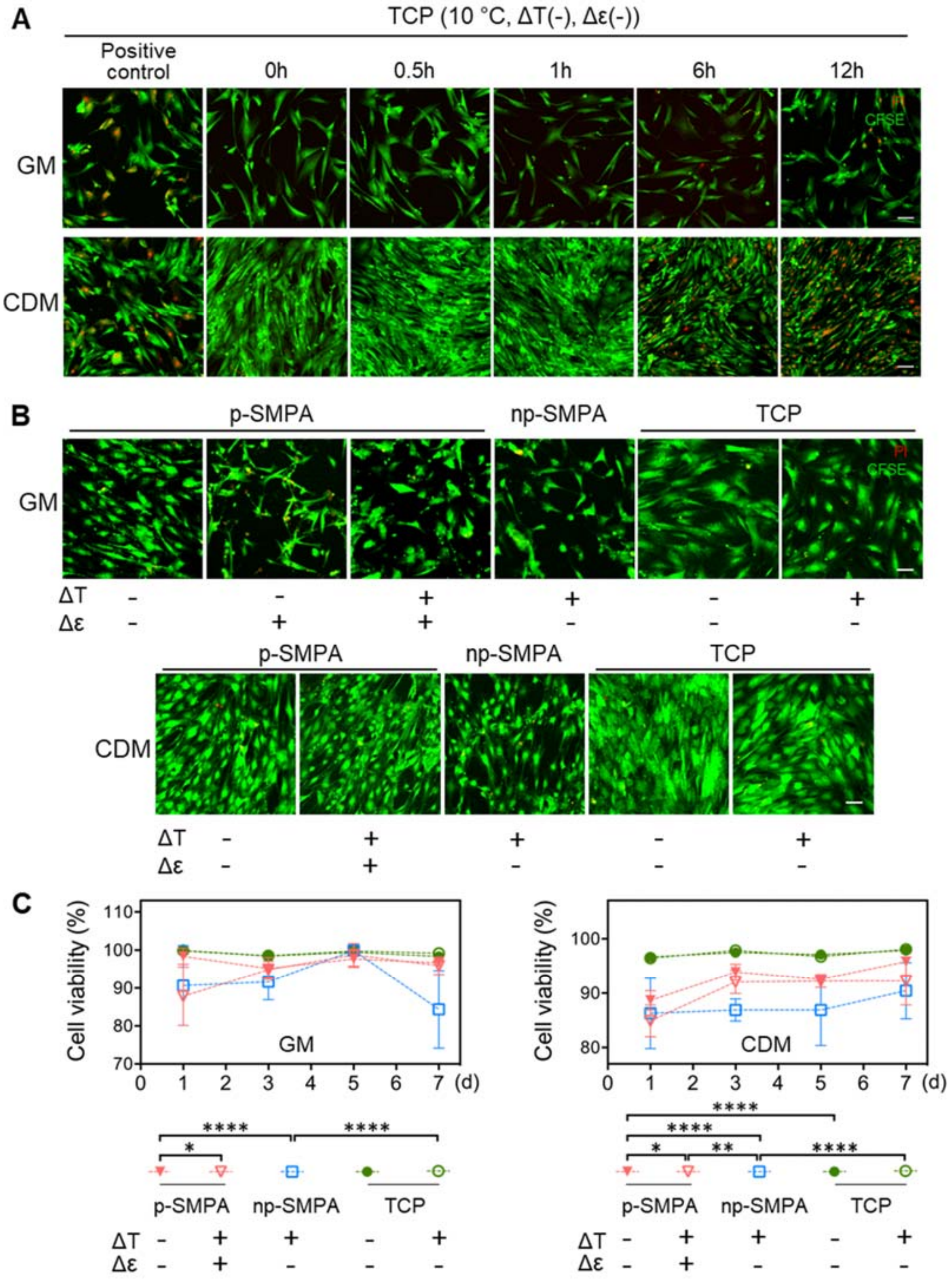
A
TCP (10 °C, ΔT(-), Δε(-))
Positive control
0h
0.5h
1h
6h
12h
GM
CDM
PI
CFSE
B
p-SMPA
np-SMPA
TCP
GM
ΔT - - + + - +
Δε - + + - - -
p-SMPA
np-SMPA
TCP
CDM
ΔT - + + - +
Δε - + - - -
C
Cell viability (%)
GM
(d)
CDM
****
*
p-SMPA
np-SMPA
TCP
ΔT - + + - +
Δε - + - - -
**

Fig. S4 (A) Live/dead staining of hADSCs cultured at 10 °C on TCP in growth medium (GM) and competitive differentiation medium (CDM). Living and dead cells were identified using CFSE (green) and propidium Iodide (red) respectively. The cells treated with sonication to disrupt the cell membrane were used as positive control (scale bar = 100 μm). (B) Live/dead staining of hADSCs cultured on SMPA sheets and TCP under different conditions (scale bar = 100 μm). (C) Quantification of cell viability using the cell viability assay kit and cytotoxicity assay kit (n >= 4; *p < 0.05, **p < 0.01, ****p < 0.0001, two-way ANOVA with Tukey's multiple comparisons test).

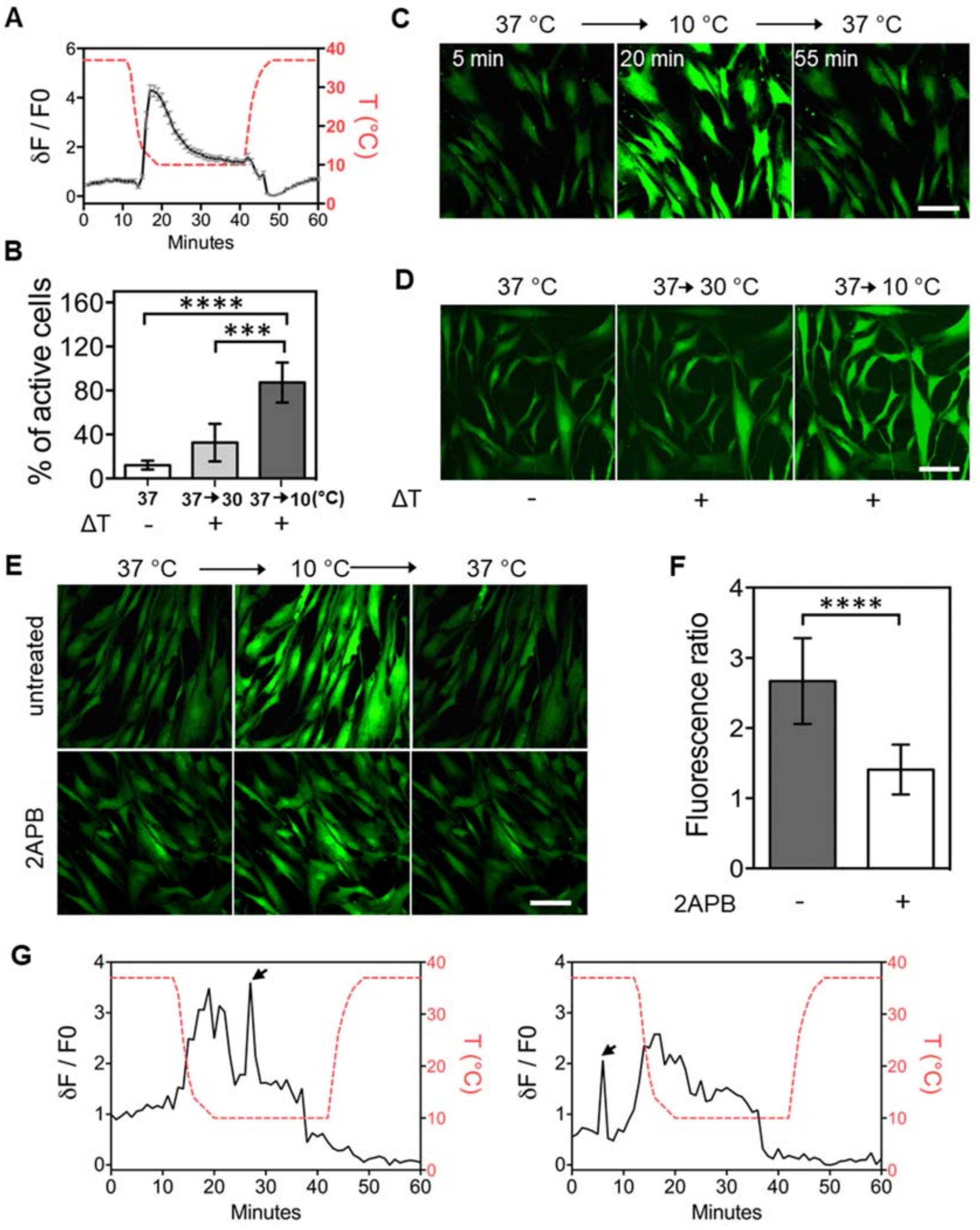

Fig. S5. (A) Dynamics of calcium fluorescence of hADSCs under cyclic temperature change. The cells were cultured on a glass coverslip in competitive differentiation medium. (B) Percentage of cells with active Ca^{2+} influx. Cells were cultured at 37 °C or in different temperature changing conditions. The analysis was based on at least 4 randomly

selected images (***p < 0.001, ****p < 0.0001, one-way ANOVA with Tukey's multiple comparisons test). (C, D) Time series images of calcium fluorescence of hADSCs cultured on glass with different switching temperatures (scale bar = 100 μm). (E) Comparison of Ca^{2+} fluorescence intensity during temperature change with and without treatment of 2APB (scale bar = 100 μm). (F) Increase of Ca^{2+} fluorescence at low temperature with and without 2APB treatment. The result was expressed as the ratio of Ca^{2+} fluorescence intensity at 10 °C to that at 37 °C. The analysis was based on more than 40 cells on 2 randomly selected images (****p < 0.0001, Student's t-test). (G) Spontaneous calcium oscillation occurred at 10 °C (left) or 37 °C (right) in hADSCs growing on p-SMPA sheets under changed temperature. The arrows indicated spontaneous calcium oscillations.

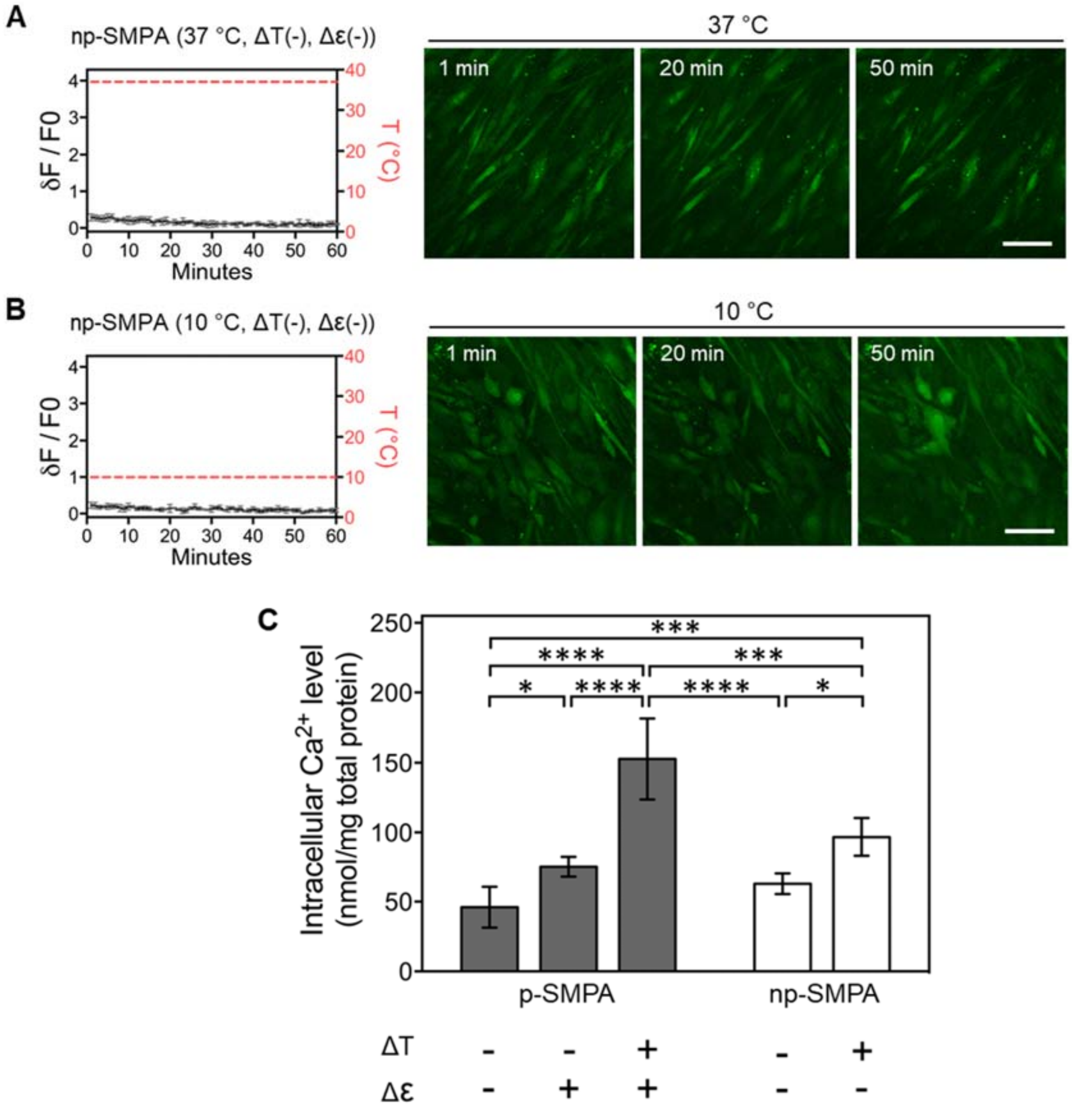

Fig. S6. Intracellular Ca^{2+} fluorescence in hADSCs without active Ca^{2+} influx on np-SMPA sheets exposed to a constant temperature of either 37 °C (A) or 10 °C (B) (n >= 3). (C) Intracellular calcium level in hADSCs cultured in competitive differentiation medium perceiving different stimuli. For ΔT-, Δε+ group, the cells were stretched using a stretching device for one cycle. For ΔT+ groups, the cells were treated with one cycle of temperature change and were lysed when the temperature reached to 10 °C. The Ca^{2+} level was determined using a calcium quantification kit (n >= 4; *$p < 0.05$, ***$p < 0.001$, ****$p < 0.0001$, one-way ANOVA with Tukey's multiple comparisons test).

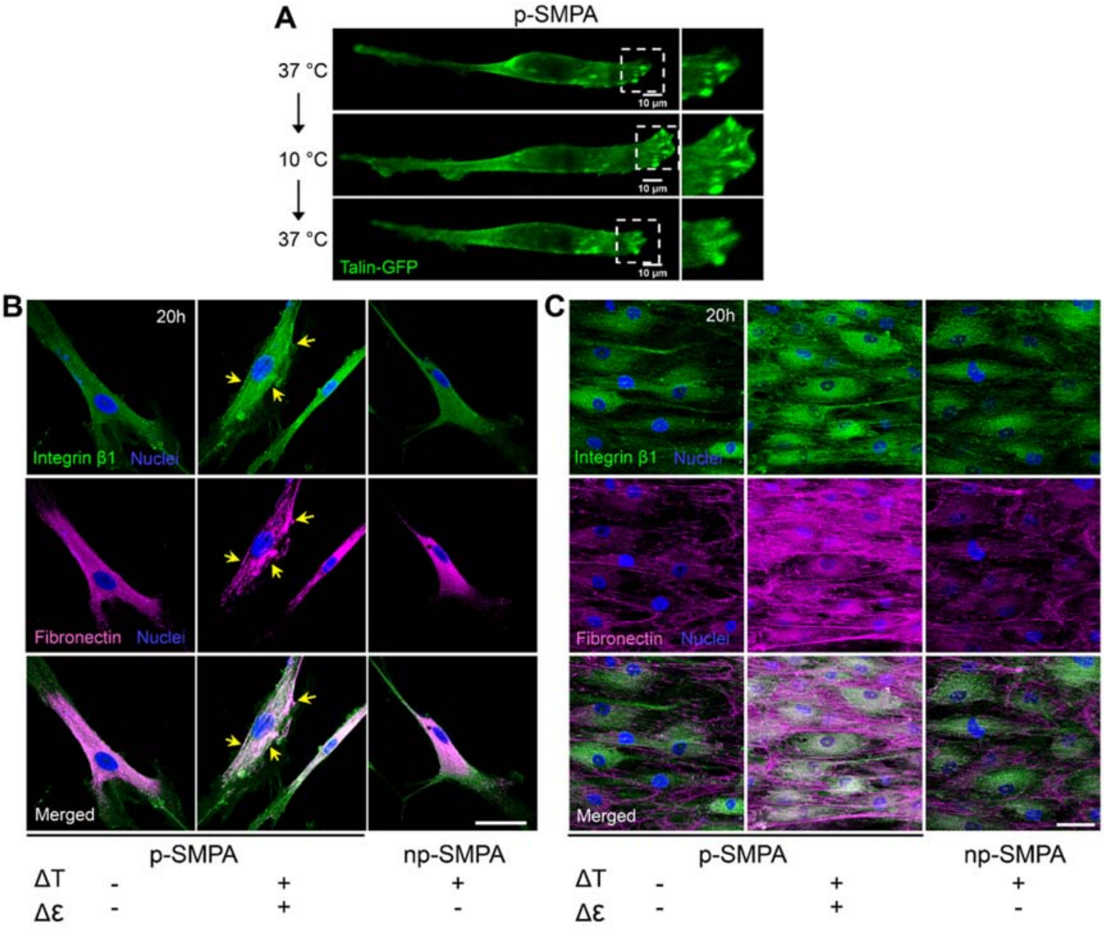

Fig. S7. (A) Representative images of talin (green) dynamics in a hADSC on p-SMPA sheet at indicated temperature. Cells were cultured in growth medium for 1 day (scale bar = 10 μm). (B, C) Fibronectin secretion and integrin β1 expression of hADSCs. The cells were seeded at low and high density to obtain the sparse (B) and confluent (C) cells on SMPA sheets. After 20 hours (20 cycles for ΔT+ groups), the cells were stained to visualize fibronectin (pink), integrin β1 (green) and cell nuclei (blue). The yellow arrows indicated the fibronectin fibrils (scale bar = 50 μm).

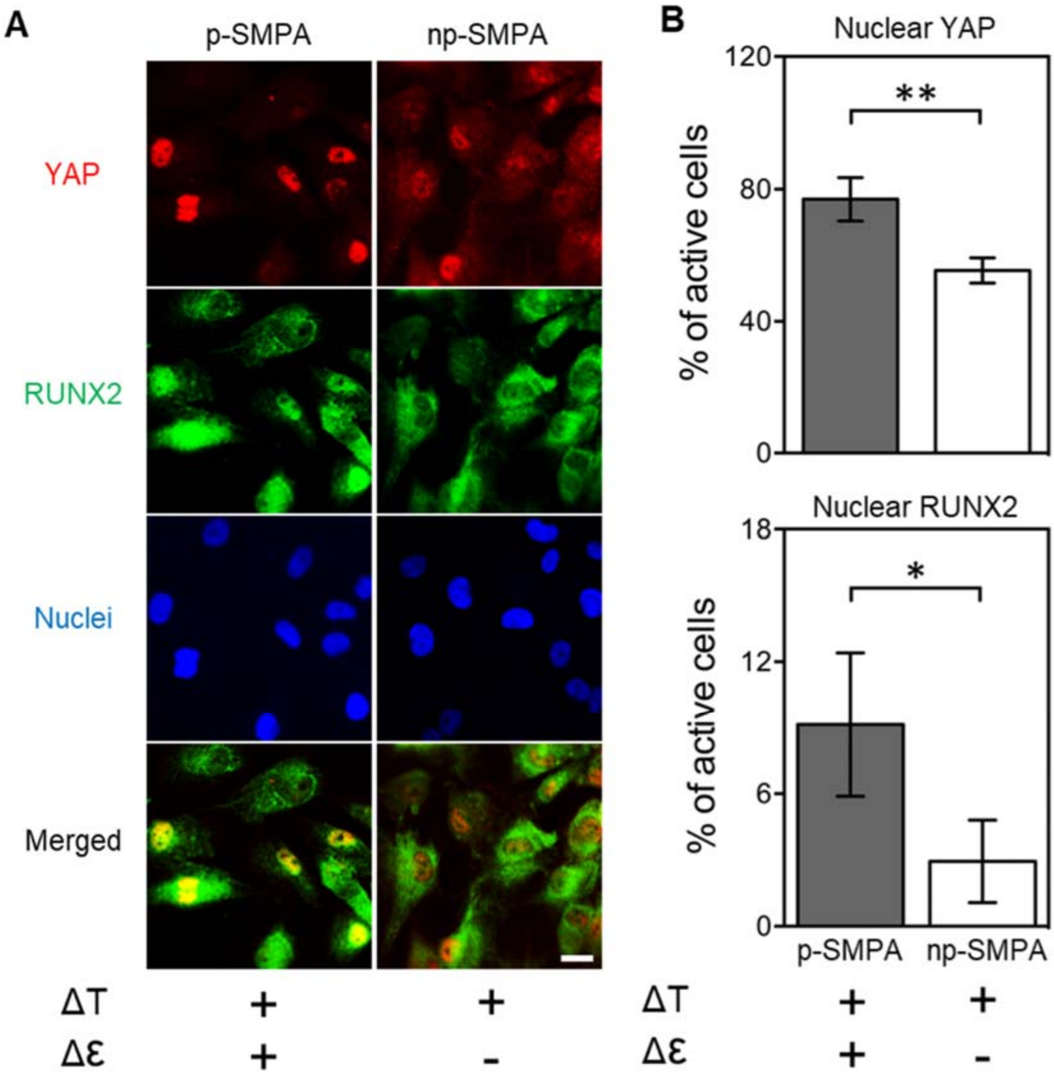

Fig. S8. (A) YAP and RUNX2 immunostaining of hADSCs on non-programmed and programmed SMPA sheets under cyclic temperature change. Cells were cultured in competitive differentiation medium for 3 days (scale bar = 20 μm). (B) Analysis of percentage of YAP and RUNX2 nuclear positive cells according to the immunostaining images. At least 3 images in each group containing more than 151 cells were analyzed (*$p < 0.05$, **$p < 0.01$, Student's *t*-test).

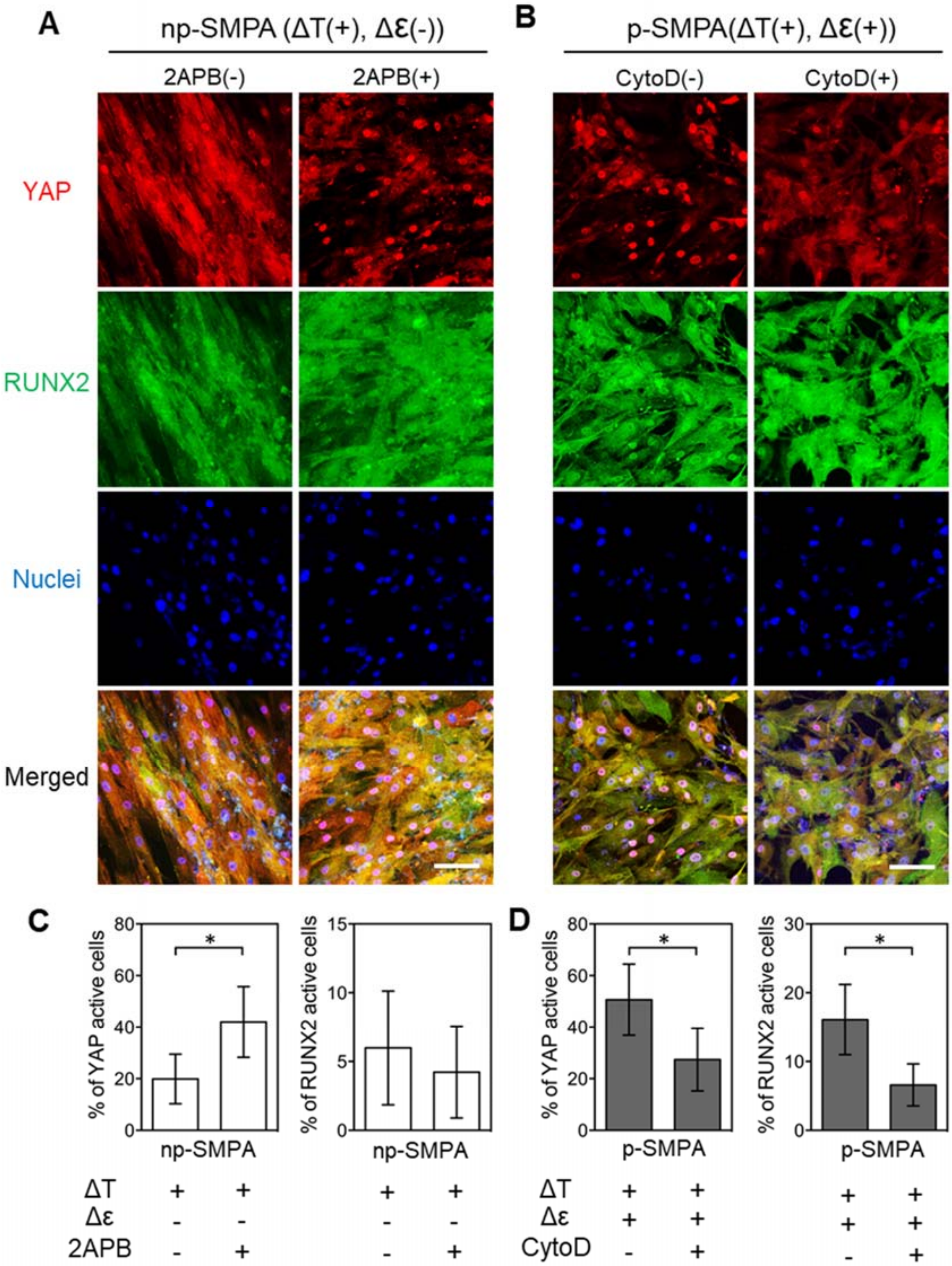
A
np-SMPA (ΔT(+), Δε(-))
2APB(-)
2APB(+)
B
p-SMPA(ΔT(+), Δε(+))
CytoD(-)
CytoD(+)
YAP
RUNX2
Nuclei
Merged
C
% of YAP active cells
% of RUNX2 active cells
np-SMPA
np-SMPA
ΔT
Δε
2APB
D
% of YAP active cells
% of RUNX2 active cells
p-SMPA
p-SMPA
ΔT
Δε
CytoD

Fig. S9 Effect of calcium signaling (A) and mechanical stimulus (B) on YAP and RUNX2 nuclear translocation. Cells cultured on SMPA sheets under different stimuli were treated with and without 2APB or CytoD for 3 hours. Cells were stained to visualize YAP (red), RUNX2 (green) and nuclei (blue) (scale bar = 100 μm). The analysis of the effect of 2APB (C) or CytoD (D) on YAP and RUNX2 activity was performed based on the fluorescence images using ImageJ software (*$p < 0.05$, Student's *t*-test). For each group, at least 4 randomly selected images were analyzed.

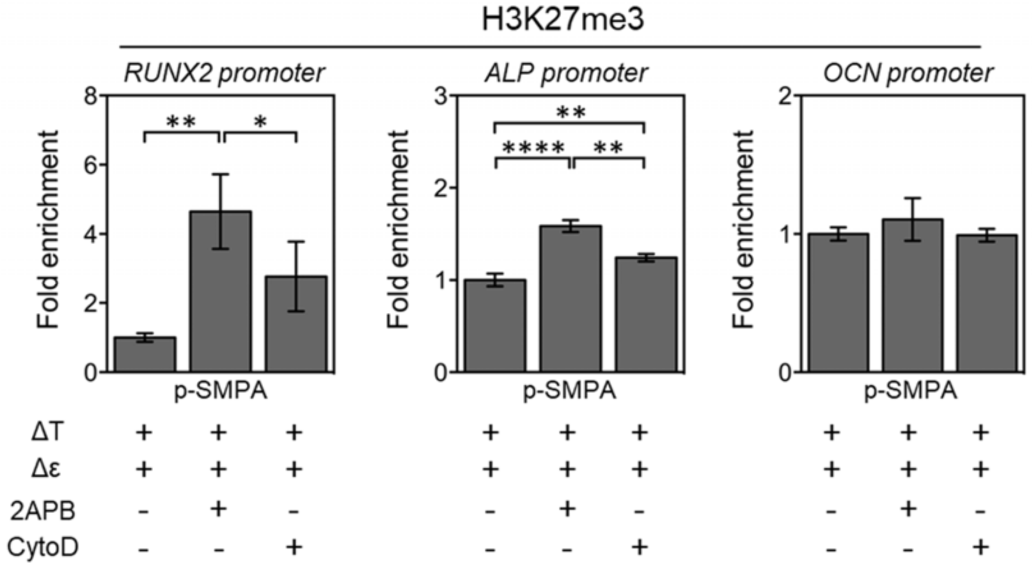

Fig. S10 Regulation of Ca^{2+} signaling and mechanical stimulus on H3K27 trimethylation on the promoters of osteogenesis related genes in hADSCs perceiving dual stimuli. 2APB and CytoD were used to inhibit the intracellular Ca^{2+} and actin polymerization. The values of the group without inhibition were set as 1 (n >= 3; *p < 0.05, **p < 0.01, ****p < 0.0001, one-way ANOVA with Tukey's multiple comparisons test).

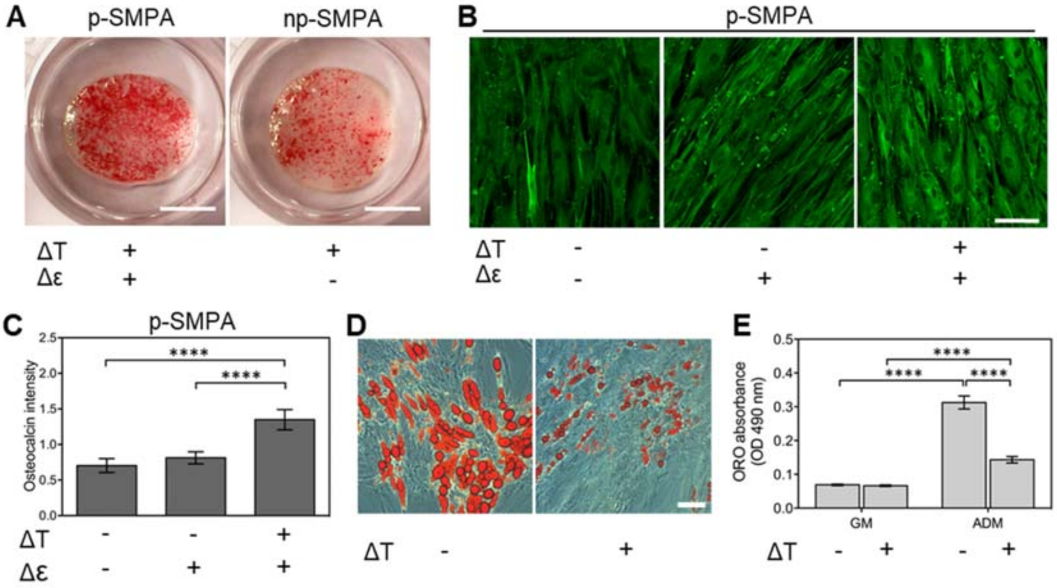

Fig. S11. (A) Alizarin Red S (ARS) staining of hADSCs cultured on programmed (p) and non-programmed (np) SMPA sheets under cyclically changed temperature. The cells were cultured in competitive differentiation medium for 3 weeks (scale bar = 0.5 cm). (B) Osteocalcin immunostaining (green) of hADSCs on p-SMPA sheet with different stimuli. Cells were cultured in competitive differentiation medium for 10 days (scale bar = 100 µm). (C) Quantification of osteocalcin fluorescence intensity according to the immunostaining images. More than 238 cells on 5 randomly selected images in each group were analyzed (****$p < 0.0001$, one-way ANOVA with Tukey's multiple comparisons test). (D) Oil Red O (ORO) staining of hADSCs induced into adipogenic differentiation. Cells were cultured on TCP in adipogenic induction medium (ADM) for 2 weeks (scale bar = 100 µm). (E) Quantification of ORO by measuring the absorbance value. Cells were cultured on TCP for 2 weeks with and without cyclic temperature change. Cells in growth medium (GM) were used as control (n = 6; ****$p < 0.0001$, one-way ANOVA with Tukey's multiple comparisons test).

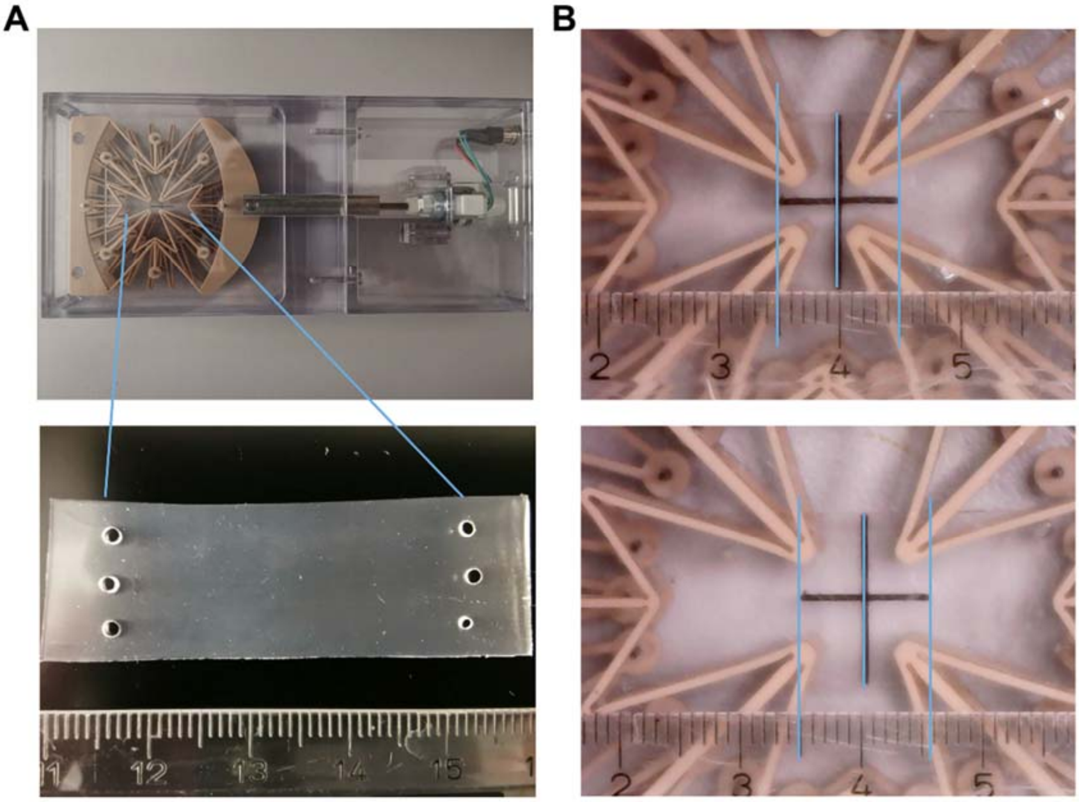

Fig. S12. (A) A stretch device was used to generate mechanical stimulus, without changing temperature. (B) Stretching of the p-SMPA sheet was along the direction of programming, resulting in an elongation about 10%.

Video S1. The p-SMPA sheets exposed to cyclic temperature change in cell growth medium. The videos were generated with the real-time images, which were obtained at 10 s intervals for 30 min using an inverted fluorescence microscopy (Axiovert 200M, Carl Zeiss, Jena, Germany). (Scale bar = 50 µm)

Video S2. The np-SMPA sheets exposed to cyclic temperature change in cell growth medium. The videos were generated with the real-time images, which were obtained at 10 s intervals for 30 min using an inverted fluorescence microscopy (Axiovert 200M, Carl Zeiss, Jena, Germany). (Scale bar = 50 µm)

Video S3. The hADSCs cultured on p-SMPA sheets exposed to cyclic temperature change in cell growth medium. The cells were stained with CFSE. The videos were generated with the real-time images, which were taken at intervals of 10 s for 30 min using an inverted fluorescence microscopy (Axiovert 200M, Carl Zeiss, Jena, Germany). (Scale bar = 50 µm)

Video S4. The hADSCs cultured on np-SMPA sheets exposed to cyclic temperature change in cell growth medium. The cells were stained with CFSE. The videos were generated with the real-time images, which were taken at intervals of 20 s for 30 min using an inverted fluorescence microscopy (Axiovert 200M, Carl Zeiss, Jena, Germany). (Scale bar = 50 µm)

Video S5. The calcium influx of hADSCs on p-SMPA sheet exposed to cyclic temperature change in competitive differentiation medium. The Ca^{2+} was probed using Fluo-4 calcium indicator. The videos were generated with the real-time images, which were taken at 5 s intervals for 18 min using a laser scanning confocal microscopy (LSM 780, Carl Zeiss, Jena, Germany). (Scale bar = 50 µm)

Video S6. The calcium influx of hADSCs on np-SMPA sheet exposed to cyclic temperature change in competitive differentiation medium. The Ca^{2+} was probed using Fluo-4 calcium indicator. The videos were generated with the real-time images, which were taken at 5 s intervals for 18 min using a laser scanning confocal microscopy (LSM 780, Carl Zeiss, Jena, Germany). (Scale bar = 50 µm)

Video S7. The calcium influx of hADSCs on glass exposed to cyclic temperature change in competitive differentiation medium. The Ca^{2+} was probed using Fluo-4 calcium indicator. The videos were generated with the real-time images, which were taken at 5 s intervals for 18 min using a laser scanning confocal microscopy (LSM 780, Carl Zeiss, Jena, Germany). (Scale bar = 50 µm)

References

1. Saatchi M, Behl M, Nöchel U, & Lendlein A (2015) Copolymer Networks From Oligo(ε-caprolactone) and n-Butyl Acrylate Enable a Reversible Bidirectional Shape-Memory Effect at Human Body Temperature. *Macromol. Rapid Commun.* 36(10):880-884.
2. Kumar UN, Kratz K, Wagermaier W, Behl M, & Lendlein A (2010) Non-contact actuation of triple-shape effect in multiphase polymer network nanocomposites in alternating magnetic field. *J. Mater. Chem.* 20(17):3404-3415.
3. Xu X*, et al.* (2014) Controlling Major Cellular Processes of Human Mesenchymal Stem Cells using Microwell Structures. *Adv Healthc Mater* 3(12):1991-2003.
